# Examining the effect of digital parenting attitudes on digital game addiction

**DOI:** 10.1590/1980-220X-REEUSP-2025-0059en

**Published:** 2025-07-28

**Authors:** Handan Kabadayı, Aslıhan Öztürk Eyimaya

**Affiliations:** 1 Ankara University, Graduate Schools of Health Sciences, Ankara, Turkey.; 2 Ankara University, Faculty of Nursing, Ankara, Turkey.

**Keywords:** Addiction, Digital, Game Addiction, Nurses, Parenting, Student, Dependência, Digital, Dependência de jogos, Enfermeiros, Parentalidade, Estudante

## Abstract

**Objective:**

This study was conducted to investigate the effect of digital parenting attitudes on digital game addiction in middle school students. Nurses can provide support to parents and students to reduce the risk of digital game addiction in children.

**Methods:**

Cross-sectional design was used in this study. The study was conducted in a middle school in X. The sample of the study consisted of 306 students and 306 parents. A “Personal Information Form,” the “Digital Parenting Attitude Scale,” and the “Digital Game Addiction Scale for Children” were used to collect data.

**Results:**

The rate of playing digital games was 83% among the students, and 24,8% played digital games for an average of an hour a day. Parents’ mean score on the “Digital Parenting Attitude Scale” was 46,97 ± 6,27. Students’ mean score on the “Digital Game Addiction Scale” was 59,42 ± 19,39. When the students were evaluated according to their digital game addiction levels, it was determined that 14,4% of them were “addicted”, 2,6% were “highly addicted”, and 54,9% were in the “at-risk” group. No significant relationship was found between the scales (p > 0,05).

**Conclusion:**

There was no significant correlation between scales. However, it was found that the majority of the students were digital game addicts or in the risk group. It is recommended that the necessary interventions be planned in cooperation with parents so that students can maintain healthy digital game playing behaviors and receive protective and preventive training to prevent digital game addiction.

## RESUMO

**Objetivo:**

Este estudo foi realizado com o objetivo de investigar o efeito das atitudes parentais digitais na dependência de jogos digitais em estudantes do ensino fundamental. Enfermeiros podem oferecer apoio a pais e alunos para reduzir o risco de dependência de jogos digitais nas crianças.

**Métodos:**

Foi utilizado um delineamento transversal neste estudo. A pesquisa foi conduzida em uma escola do ensino fundamental em X. A amostra do estudo foi composta por 306 alunos e 306 pais. Foram utilizados um “Formulário de Informações Pessoais”, a “Escala de Atitudes Parentais Digitais” e a “Escala de Dependência de Jogos Digitais para Crianças” para coleta de dados.

**Resultados:**

A taxa de alunos que jogavam jogos digitais era de 83%, sendo que 24,8% jogavam em média uma hora por dia. A pontuação média dos pais na “Escala de Atitudes Parentais Digitais” foi de 46,97 ± 6,27. A pontuação média dos alunos na “Escala de Dependência de Jogos Digitais” foi de 59,42 ± 19,39. Ao avaliar os alunos de acordo com seus níveis de dependência de jogos digitais, observou-se que 14,4% eram “dependentes”, 2,6% “altamente dependentes” e 54,9% estavam no grupo “em risco”. Não foi encontrada relação significativa entre as escalas (p > 0,05).

**Conclusão:**

Não foi encontrada correlação significativa entre as escalas. No entanto, observou-se que a maioria dos alunos era dependente de jogos digitais ou estava no grupo de risco. Recomenda-se que sejam planejadas intervenções necessárias em cooperação com os pais, para que os alunos possam manter comportamentos saudáveis relacionados aos jogos digitais e recebam educação preventiva e protetiva contra a dependência de jogos digitais.

## INTRODUCTION

Digital games have become one of the popular sources of entertainment and activity for students today. Excessive and uncontrolled game-playing habits can turn into digital game addiction. Digital game addiction can have negative effects on students’ physical, social, and emotional health and impact their daily lives^(1,2)^. For these reasons, the attitudes taken by parents to balance and control digital game use have an important role in determining the risk of game addiction in their children. In digital game addiction, playing games becomes a goal for the individual. The individual isolates himself/herself from the real world, develops tolerance to playing games, and shows withdrawal symptoms when he/she does not play games, just like in other types of addiction^(3)^.

The necessity of preventing digital game addiction and raising awareness among parents about the correct use of technology has led to the emergence of the concept of “digital parenting”^(1)^. Digital parenting aims to support children in using digital technologies correctly while protecting them from online risks and helping them develop their social, emotional, and cognitive skills in the digital world. In this context, parents inform and guide their children about digital technology use and online behaviors^(3)^. Digital parenting attitudes include parents’ perceptions, approaches, and behaviors about how their children will use digital technologies. Parental attitudes toward children’s digital game use can affect their game-playing habits and reduce or increase the risk of digital game addiction^(4)^. Parents’ supervision and guidance of their children’s digital game use plays an important role in reducing the risk of digital game addiction. When parents set consistent rules for the selection of games and determine durations, this helps children regulate their gaming habits. In addition, setting consistent rules encourages children to share their concerns and problems about games with their parents and helps to detect possible addiction symptoms early. Digital parenting attitudes shape children’s digital game use and thus determine the risk of digital game addiction^(4,5)^.Some studies conducted to reveal the relationship between digital parenting attitudes and digital game addiction have shown that parents’ attitudes can influence children’s gaming habits^(3,4)^. In addition, it has been stated in the literature that the time children spend on digital tools is directly proportional to the time parents spend^(6)^.

To prevent digital game addiction, children should be provided with training from an early age on the correct use of digital technologies, risks, and determining appropriate time and content. Nurses can provide support for parents and students to reduce the risk of digital game addiction in children. They can provide training and consultancy for parents on digital parenting. They can guide parents on setting limits on their children’s digital game playing, raising awareness about safe online behaviors, and monitoring their children’s gaming habits^(2,7)^. With the education and counseling provided by nurses, parents can develop more effective strategies to balance their children’s digital game use and reduce the risk of addiction. In addition, they can educate students about the effects of digital games. They can increase students’ awareness of this issue by organizing awareness programs about digital game addiction^(2,8,9)^. They can support students who show signs of digital game addiction by holding individual interviews with them and ensuring that they get professional help when necessary. They can also teach students preventive strategies such as time management and stress-coping skills. With the education, support, and guidance they provide for parents and students, nurses can help children develop healthy digital technology use habits and reduce the risk of digital game addiction. Nurses should take an active role in digital game addiction and support children’s digital technology use habits^(8,10)^. Digital game addiction is common among students. Digital game addiction can cause problems such as deterioration in social interaction and communication. Nurses can assess students’ gaming behaviors. Nurses should develop strategies to prevent digital game addiction^(11)^. Digital parenting attitudes should be taken into consideration when examining digital game addiction in students. Nurses should organize training programs to prevent digital game addiction. Parents’ digital attitudes play an important role in shaping children’s relationships with digital games. In this context, parents’ positive or negative approaches to digital media use can directly affect children’s risk of developing game addiction. Parents’ low levels of digital awareness can cause children to spend uncontrolled time in front of the screen. Overly controlling or completely disinterested digital attitudes can also trigger addictive behaviors in children. This can negatively affect children’s psychosocial development, academic success, and physical health. School health nurses play a critical role in monitoring and guiding children’s digital gaming habits. Nurses can support children in gaining healthy digital habits by informing parents about their digital attitudes. They can organize educational programs for digital media use in collaboration with families and raise awareness about early signs and risk factors related to game addiction. They can also provide individual counseling for students at risk of game addiction and refer them to relevant health professionals when necessary. In this context, it is of great importance for school health nurses to take on both a preventive and supportive role in the fight against digital addiction^(11,12,13)^.

There are some studies in the literature on digital game addiction and parenting attitudes^(6,14,15)^. However, there are no studies on the impact of digital parenting attitudes on digital game addiction in middle school students. This study is original in that it was conducted to investigate the effect of digital parenting attitudes on digital game addiction in middle school students. Therefore, it is thought that the results of the research will contribute to the literature.

The research questions are as follows:

1. What is the digital game addiction level of students?
2. What is the level of parents’ digital parenting attitudes?
3. Is there a significant difference between students’ descriptive characteristics and the mean “Digital Game Addiction Scale for Children” scores?
4. Is there a significant difference between parents’ descriptive characteristics and the mean “Digital Game Addiction Scale for Children” scores?
5. Is there a significant correlation between the mean “Digital Parenting Attitude Scale” and the “Digital Game Addiction Scale for Children” scores?

## METHOD

### Design and Participants

A cross-sectional design was employed. This research was conducted to investigate the effect of digital parenting attitudes on digital game addiction in middle school students attending a middle school located in X. The research data were collected between December 2023 and February 2024. According to the Socio-Economic Development Ranking Research 2022, which aimed to measure the socio-economic development level of districts in our country on a district basis, Mamak district has a second-grade development level. It is one of the centers receiving migration from other settlements. Districts at this level provide employment in the manufacturing industry and service sectors. The basic infrastructure of these districts has almost been completed, and the quality of education, health, and life is high compared to many districts^(16)^. Since all public schools in our country receive the same funding from the government, the school in Mamak district where the study would be conducted was selected by drawing lots, without applying a socio-economical classification procedure.

There were 1,511 students registered at the school on the data collection dates. So, the population of the study consisted of 1,511 secondary school students and 1,511 parents. The sampling of the known population formula was employed to determine the sample size for the study^(17)^. According to the formula, at least 306 students and 306 parents were included in the sample. The number of students to be sampled from each branch was determined by calculating their proportions to the general population. The students were divided into four strata according to their grades (grades 5, 6, 7, and 8). The number of students to be included in the sample was determined with proportional selection from each stratum according to the number of students in the strata, and students from each stratum were selected by simple random sampling method. All students in the branches were listed and numbered by using class lists. Then, the students to be included in the sample were determined using a random number table. The selection process continued until the determined sample size was reached from each branch. The sample included 83 students from the fifth grade, 74 from the sixth grade, 79 from the seventh grade, and 70 from the eighth grade.

Inclusion criteria were age between 10 and 14 years, parents of students in this age range, and agreeing to participate in the study voluntarily. Exclusion criteria were visual or hearing impairment or a neuropsychiatric disease in the student or the parent, or delivering an incomplete data collection form.

### Instruments

Data collection instruments included a personal information form for students and parents, the Digital Parenting Attitude Scale, and the Digital Game Addiction Scale for Children.

### The Personal Information Form

This form was prepared by the researchers following a review of the literature. It included questions about the student’s age, gender, grade, digital game-playing status, and the digital game- playing status of their parents and about parents’ age, gender, education status, occupation, socioeconomic level, and how long they used technological devices a day^(18,19)^.

### The Digital Parenting Attitude Scale (DPAS)

This tool was developed by İnan Kaya et al.^(4)^ to determine the attitudes of parents toward the use of digital media tools by their children. It consists of 12 items used to measure the digital parenting attitudes of parents with children aged 6–18. The scale consists of two sub-dimensions: “approval of effective use of digital media” and “protection from digital media risks.” A five-point Likert-type scale is used to evaluate the items on the scale. Scores on the scale range from 12 to 60. Cronbach’s alpha coefficient of the sub-dimensions is 0.77 and 0.72, respectively. The alpha coefficients for the sub-dimensions were found as 0.82 and 0.86, respectively. The alpha value of the total DPAS was estimated to be 0.87.

### The Digital Game Addiction Scale for Children (DGASC)

This scale was developed by Hazar and Hazar to determine the level of digital game addiction in children between the ages of 10 and 14^(20)^. It consists of 24 items and four sub-dimensions: “tolerance development during playtime and the value placed on play,” “excessive focus on playing digital games and conflict,” “postponement of individual and social duties/tasks,” “psychological-physiological reflection of deprivation and immersion in play.” A five-point Likert-type scale was used to evaluate the items on the scale (1 = disagree, 2 = disagree, 3 = undecided, 4 = agree, 5 = completely agree). The lowest score on the scale is 24 and the highest is 120. Scores are interpreted as follows: 1–24: normal group; 25–48: low-risk group; 49–72: risky group; 73–96: addicted group; 97–120: highly addicted group. High scores on the scale indicate a high level of digital game addiction. Cronbach’s alpha coefficient is .78 for the first sub-factor, .81 for the second sub-factor, .76 for the third sub-factor, .67 for the fourth sub-factor, and .90 for the total scale. In this thesis study, Cronbach’s alpha coefficient was found to be 0.96 for the total scale and 0.90, 0.88, 0.90, and 0.80 for sub-factors, respectively.

### Data Collection

The school administration was informed about the research, and the classes where the application would be carried out and and dates were determined with the psychological counseling and guidance teachers. An informed consent form was sent to the parents of the students who would participate in the study in a sealed envelope and was collected from the students by teachers within a week. The classes were visited in the company of psychological counseling and guidance teachers, the students were informed about the study, and verbal consent was obtained from those who agreed to participate in the study. Data collection tools were distributed to the students and they were asked to fill them out under the supervision of the researcher. The questionnaires that parents who agreed to participate in the study would fill out were delivered to the students to take to their parents, and the data were collected. It took approximately 15 minutes to fill out the scales.

### Ethical Consideration

To carry out the study, the approval of the XX Ethics Committee (date: 20/06/2023 and number: 11/88) and the institutional permission of the XX (date: 29/09/2023 and number: 1093612) were obtained. Verbal consent was obtained from the students, and written consent was obtained from the parents included in the study.

### Data Analysis

Data were analyzed on the SPSS for Windows 22 software package. Numbers, percentages, minimum and maximum values, mean, and standard deviation figures were used in data analysis. The normality assumption of the data was calculated with the “Kurtosis” and “Skewness” coefficients (±2)^(21)^.All data were found to have a normal distribution. Independent groups t-test and variance analysis (LSD in cases where variances were homogeneous as advanced analysis and Dunnet C in cases where they were not) were used in the comparison of paired groups. Pearson correlation analysis was used for relational inferences.

## RESULTS

Parents’ mean scores were 22.88 ± 3.54 on the approval of effective use of digital media sub-dimension, 24.09 ± 3.70 on the protection from digital media risks sub-dimension, and 46.97 ± 6.27 on total Digital Parenting Attitude Scale (Table 1).

**Table 1:** Distribution of scores from the Digital Parenting Attitude Scale and its sub-dimensions (n = 306) – Ankara, Turkey, 2024.

Scale and subscales	Min.	Max.	Mean	SD
Approval of effective use of digital media	10.00	30.00	22.88	3.54
Protection from digital media risks	12.00	30.00	24.09	3.70
Digital Parenting Attitude Scale	22.00	60.00	46.97	6.27

Children’s mean scores from the Digital Game Addiction Scale were as follows: 18.99 ± 5.97 on the tolerance development during playtime and the value placed on play sub-dimensions; 17.53 ± 6.13 on the excessive focus on playing digital games and conflict sub-dimension; 13.15 ± 5.00 on the postponement of individual and social duties/tasks sub-dimension; 9.75 ± 3.57 on the psychological-physiological reflection of deprivation and immersion in play sub-dimension; and 59.42 ± 19.39 on the total scale. According to the cut-off points of the scale, 14.4% of the students were addicted, 2.6% were highly addicted, and 54.9% were in the at-risk group (Table 2).

**Table 2:** Distribution of scores obtained from the Digital Game Addiction Scale for Children and its sub-dimensions – Ankara, Turkey, 2024.

Scale and subscales	n	Min.	Max.	Mean	SD
Tolerance development during playtime and the value placed on play	306	7.00	35.00	18.99	5.97
Excessive focus on playing digital games and conflict	306	7.00	35.00	17.53	6.13
Postponement of individual and social duties/tasks	306	6.00	30.00	13.15	5.00
Psychological-physiological reflection of deprivation and immersion in play	306	4.00	20.00	9.75	3.57
Digital Game Addiction Scale for Children	306	24.00	120.00	59.42	19.39
Cut-off point distribution	n	%	–	–	–
Normal	12	3.9	–	–	–
Low risk	74	24.2	–	–	–
Risky	168	54.9	–	–	–
Addicted	44	14.4	–	–	–
Highly addicted	8	2.6	–	–	–

The difference between students’ mean scores on the “tolerance development during playtime and the value placed on play” and “psychological-physiological reflection of deprivation and immersion in play” sub-dimensions and the total DGASC were found to be statistically significant according to the status of absenteeism from school to play digital games. The mean scores of students who did not skip school were higher (p < 0.05). The difference between the mean postponement of individual and social duties/tasks sub-dimension scores was statistically significant according to students’ grades (p < 0.05). The mean scores of the 8^th^ graders were higher than those of the 6^th^ graders (Table 3).

**Table 3:** Comparison of students' scores from DGASC and its sub-dimensions according to their descriptive characteristics – Ankara, Turkey, 2024.

Sociodemographic characteristics		n (%)	Tolerance development during playtime and the value placed on play		Excessive focus on playing digital games and conflict		Postponement of individual and social duties/tasks		Psychological-physiological reflection of deprivation and immersion in play		DGASC	
			Mean	SD	Mean	SD	Mean	SD	Mean	SD	Mean	SD
Age	10	41 (13.4)	18.73	5.66	17.98	5.58	12.66	4.59	9.83	3.31	59.20	18.00
	11	61 (19.9)	18.28	5.06	16.87	5.42	11.97	3.77	9.26	3.29	56.38	16.36
	12	89 (29.1)	18.81	6.81	17.38	6.92	13.33	5.56	9.62	3.69	59.13	21.66
	13	76 (24.9)	19.24	5.72	17.83	6.08	13.58	5.23	9.95	3.80	60.59	19.55
	14	39 (12.7)	20.31	6.06	17.82	6.10	14.28	5.12	10.38	3.57	62.79	19.63
	Test and significance		F = 0.762 p = 0.551		F = 0.309 p = 0.872		F = 1.631 p = 0.166		F = 0.683 p = 0.604		F = 0.744 p = 0.563	
Gender	Male	113 (36.9)	19.37	5.97	17.91	6.19	13.04	5.17	9.79	3.70	60.12	19.48
	Female	193 (63.1)	18.77	5.97	17.30	6.09	13.21	4.91	9.74	3.50	59.02	19.38
	Test and significance		t = 0.855 p = 0.393		t = 0.841 p = 0.401		t = -0.283 p = 0.777		t = 0.122 p = 0.903		t = 0.478 p = 0.633	
Grade	5	83 (27.1)	18.83	5.75	17.80	5.95	12.54	4.62	9.75	3.50	58.92	18.80
	6	74 (24.2)	18.01	5.76	16.55	6.16	12.24	4.66	9.11	3.49	55.92	18.62
	7	79 (25.8)	19.11	6.37	17.66	6.20	13.37	5.19	9.75	3.59	59.89	19.98
	8	70 (22.9)	20.07	5.90	18.09	6.23	14.59	5.33	10.46	3.67	63.20	19.91
	Test and significance		F = 1.465 p = 0.224		F = 0.880 p = 0.452		F = 3.261 p = 0.022		F = 1.725 p = 0.162		F = 1.737 p = 0.159	
Having a personal computer/tablet	Yes	113 (36.9)	18.69	5.66	17.36	5.71	13.04	4.83	9.35	3.20	58.44	18.04
	No	193 (63.1)	19.17	6.15	17.62	6.37	13.22	5.11	9.99	3.76	59.99	20.17
	Test and significance		t = −0.672 p = 0.502		t = −0.356 p = 0.722		t = −0.307 p = 0.759		t = −1.571 p = 0.117		t = −0.675 p = 0.500	
Having a personal phone	Yes	82 (26.8)	17.99	5.89	16.78	5.87	12.94	4.75	9.10	3.35	56.80	18.57
	No	224 (73.2)	19.36	5.97	17.80	6.21	13.23	5.10	10.00	3.63	60.38	19.64
	Test and significance		t = −1.784 p = 0.075		t = −1.289 p = 0.198		t = −0.446 p = 0.656		t = −1.958 p = 0.051		t = −1.430 p = 0.154	
Having internet connection at home	Yes	276 (90.2)	18.91	5.74	17.41	5.99	13.11	4.89	9.71	3.52	59.14	18.81
	No	30 (9.8)	19.70	7.81	18.63	7.28	13.50	6.01	10.17	4.06	62.00	24.32
	Test and significance		t = −0.536 p = 0.595		t = −1.042 p = 0.298		t = −0.403 p = 0.688		t = −0.665 p = 0.507		t = −0.766 p = 0.444	
Status of playing digital games	Yes	254 (83.0)	18.91	5.83	17.37	6.00	13.04	4.89	9.65	3.47	58.96	18.81
	No	52 (17.0)	19.37	6.66	18.31	6.71	13.71	5.54	10.27	4.04	61.65	22.09
	Test and significance		t = −0.497 p = 0.620		t = −1.009 p = 0.314		t = −0.888 p = 0.375		t = −1.141 p = 0.255		t = −0.911 p = 0.363	
The device on which digital games are played most	Computer/Tablet	174 (56.9)	18.89	5.52	17.41	5.77	13.02	4.77	9.76	3.44	59.09	18.19
	Mobile Phone	86 (28.1)	19.34	6.61	17.94	6.66	13.38	5.42	9.69	3.70	60.35	21.02
	Game Console	8 (2.6)	17.88	3.83	16.00	4.69	11.88	2.47	9.75	3.15	55.50	13.14
	None	38 (12.4)	18.92	6.86	17.42	6.85	13.47	5.54	9.87	4.04	59.68	22.34
	Test and significance		F = 0.208 p = 0.891		F = 0.318 p = 0.812		F = 0.324 p = 0.808		F = 0.024 p = 0.995		F = 0.193 p = 0.901	
Daily digital game playing time	None	31 (10.1)	19.32	6.98	17.84	6.95	13.58	5.45	10.23	4.03	60.97	22.51
	0–15 minutes	17 (5.6)	19.59	6.96	18.29	7.46	13.82	6.76	9.59	4.57	61.29	25.24
	15–30 minutes	25 (8.2)	17.12	7.19	16.04	7.13	11.76	5.74	8.92	3.48	53.84	22.15
	30 minutes– 1 hour	50 (16.3)	18.50	6.34	17.30	6.64	13.40	5.38	9.56	3.75	58.76	21.02
	1 hour	76 (24.8)	19.32	4.91	17.84	5.31	13.29	4.53	10.07	3.42	60.51	16.66
	2 hours	64 (20.9)	18.97	5.87	16.98	6.12	12.73	4.72	9.67	3.69	58.36	18.98
	3 hours	27 (8.8)	18.41	3.65	17.44	4.01	12.63	3.41	9.52	2.55	58.00	12.16
	≥4 hours	16 (5.2)	21.69	7.51	19.94	6.56	14.88	5.33	10.19	3.10	66.69	20.92
	Test and significance		F = 0.973 p = 0.451		F = 0.720 p = 0.655		F = 0.751 p = 0.629		F = 0.431 p = 0.882		F = 0.754 p = 0.626	
Absenteeism from school to play digital games	Yes	5 (1.6)	13.40	2.61	12.20	4.15	10.40	4.34	6.40	1.14	42.40	10.36
	No	301 (98.4)	19.08	5.96	17.61	6.12	13.20	5.01	9.81	3.57	59.70	19.39
	Test and significance		t = −2.124 p = 0.034		t = −1.969 p = 0.050		t = −1.241 p = 0.216		t = −6.203 p = 0.001		t = −1.988 p = 0.048	
Whether the mother plays digital games	Yes	78 (25.5)	20.03	5.48	18.26	5.67	13.50	4.40	10.29	3.19	62.08	17.54
	No	228 (74.5)	18.64	6.10	17.28	6.27	13.03	5.20	9.57	3.68	58.51	19.94
	Test and significance		t = 1.782 p = 0.076		t = 1.220 p = 0.223		t = 0.715 p = 0.475		t = 1.551 p = 0.122		t = 1.403 p = 0.162	
Whether the father plays digital games	Yes	87 (28.4)	18.60	5.76	16.74	5.24	12.39	4.03	9.43	3.29	57.15	16.75
	No	219 (71.6)	19.15	6.05	17.84	6.43	13.45	5.32	9.89	3.67	60.32	20.32
	Test and significance		t = −0.725 p = 0.469		t = −1.425 p = 0.155		t = −1.888 p = 0.060		t = −1.018 p = 0.309		t = −1.293 p = 0.197	
Whether the mother or father is aware of the content of the digital games that the child plays	Yes	144 (47.1)	18.96	6.09	17.57	6.10	13.21	5.15	9.73	3.61	59.47	19.60
	No	162 (52.9)	19.02	5.87	17.49	6.18	13.10	4.89	9.78	3.54	59.38	19.27
	Test and significance		t = −0.088 p = 0.930		t = 0.116 p = 0.907		t = 0.191 p = 0.849		t = −0.119 p = 0.906		t = 0.037 p = 0.970	
Whether the mother or father plays digital games with the child	Yes	44 (14.4)	19.95	5.49	18.27	5.96	13.66	5.39	10.43	3.66	62.32	19.44
	No	262 (85.6)	18.83	6.04	17.40	6.16	13.06	4.94	9.64	3.55	58.94	19.38
	Test and significance		t = 1.159 p = 0.247		t = 0.873 p = 0.383		t = 0.728 p = 0.467		t = 1.361 p = 0.174		t = 1.071 p = 0.285	
Whether the mother or father supervises digital game playing	Yes	195 (63.7)	18.62	5.78	17.23	6.06	12.93	5.03	9.70	3.71	58.48	19.33
	No	111 (36.3)	19.65	6.26	18.05	6.25	13.53	4.95	9.85	3.33	61.07	19.48
	Test and significance		t = −1.459 p = 0.146		t = −1.118 p = 0.264		t = −1.006 p = 0.315		t = −0.339 p = 0.735		t = −1.124 p = 0.262	
Considering yourself a digital game addict	Yes	6 (2.0)	21.33	9.79	19.00	8.07	14.83	6.82	9.00	2.97	64.17	25.90
	Partly	43 (14.0)	18.72	5.37	17.98	5.68	13.79	4.51	9.98	3.58	60.47	17.97
	No	257 (84.0)	18.98	5.97	17.42	6.17	13.00	5.04	9.74	3.59	59.14	19.53
	Test and significance		F = 0.505 p = 0.604		F = 0.330 p = 0.719		F = 0.801 p = 0.450		F = 0.220 p = 0.803		F = 0.268 p = 0.765	

The difference between children’s scores on tolerance development during playtime and the value placed on play, excessive focus on playing digital games and conflict, and postponement of individual and social duties/tasks sub-dimensions and the total DGASC was statistically significant according to parents’ gender. The sub-dimension and total mean scores of the children of female parents were significantly higher (p < 0.05). The difference between students’ mean scores on excessive focus on playing digital games and conflict, postponement of individual and social duties/tasks, and psychological-physiological reflection of deprivation and immersion in play sub-dimensions and the total DGASC was statistically significant according to the frequency of parents’ use of technological devices and the technological devices they used. In the further analysis performed to determine which group caused the difference in terms of frequency of use of technological devices, it was determined that the mean scale scores of the children of those who used technological devices several times a month were higher than those of the other groups (p < 0.05) (Table 4).

**Table 4:** Comparison of DGASC and sub-dimension scores according to parents’ demographic characteristics – Ankara, Turkey, 2024.

Sociodemographic characteristics		n (%)	Tolerance development during playtime and the value placed on play		Excessive focus on playing digital games and conflict		Postponement of individual and social duties/tasks		Psychological-physiological reflection of deprivation and immersion in play		DGASC	
			Mean	SD	Mean	SD	Mean	SD	Mean	SD	Mean	SD
Gender	Male	99 (32.4)	17.71	5.98	16.36	5.94	12.31	4.47	9.27	3.23	55.66	18.43
	Female	207 (67.6)	19.60	5.88	18.08	6.15	13.55	5.20	9.99	3.71	61.22	19.63
	Test and significance		t = −2.626 p = 0.009		t = −2.311 p = 0.021		t = -2.035 p = 0.043		t = -1.639 p = 0.102		t = -2.366 p = 0.019	
Age	25–29	2 (0.7)	14.50	3.54	15.00	4.24	9.00	4.24	7.00	1.41	45.50	13.44
	30–34	65 (21.2)	20.74	5.79	19.52	5.82	14.25	5.01	10.86	3.42	65.37	18.89
	35–39	119 (38.9)	18.73	5.84	17.38	6.25	12.99	4.91	9.75	3.73	58.85	19.59
	40–44	73 (23.8)	18.51	6.05	16.70	6.11	13.00	5.26	9.25	3.47	57.45	19.37
	45–49	32 (10.5)	17.97	5.14	16.44	4.99	12.50	3.93	9.06	3.19	55.97	15.64
	49+	15 (4.9)	18.60	8.09	16.73	7.67	12.33	6.31	9.33	3.62	57.00	24.43
	Test and significance		F = 1.703 p = 0.134		F = 2.014 p = 0.077		F = 1.127 p = 0.346		F = 2.104 p = 0.065		F = 1.876 p = 0.098	
Family type	Extended	31 (10.1)	18.65	5.90	16.94	5.43	12.29	3.22	9.58	3.49	57.45	16.81
	Core	265 (86.6)	19.03	5.95	17.61	6.18	13.25	5.13	9.78	3.57	59.67	19.57
	Broken	10 (3.3)	19.10	7.17	17.10	7.25	13.10	6.26	9.60	4.22	58.90	23.53
	Test and significance		F = 0.058 p = 0.944		F = 0.193 p = 0.825		F = 0.513 p = 0.599		F = 0.053 p = 0.948		F = 0.185 p = 0.832	
Education	Non-literate	5 (1.6)	19.00	6.48	18.00	6.96	13.60	6.54	9.80	3.90	60.40	23.42
	Elementary school	46 (15.0)	19.80	5.08	17.54	5.30	13.46	3.87	9.59	3.36	60.39	15.83
	Middle school	57 (18.6)	19.30	6.18	17.91	6.67	13.05	5.35	9.89	3.48	60.16	20.42
	High school	119 (38.9)	18.71	6.29	17.34	6.29	13.13	4.95	9.81	3.80	58.97	20.14
	Undergraduate	75 (24.5)	18.57	5.87	17.59	6.16	13.05	5.55	9.68	3.54	58.89	20.10
	Postgraduate	4 (1.3)	21.50	5.57	15.75	0.50	13.00	2.16	9.50	1.00	59.75	2.99
	Test and significance		F = 0.466 p = 0.801		F = 0.141 p = 0.983		F = 0.053 p = 0.998		F = 0.053 p = 0.998		F = 0.065 p = 0.997	
Number of children	1	62 (20.3)	18.42	6.07	17.71	6.17	12.60	5.34	9.71	3.80	58.44	20.21
	2	164 (53.6)	19.18	5.67	17.68	6.04	13.51	4.86	9.87	3.51	60.23	18.73
	3	57 (18.6)	18.39	7.03	16.44	6.86	12.63	5.43	9.40	3.87	56.86	22.01
	4	21 (6.9)	21.29	4.28	19.14	4.22	13.81	3.71	10.24	2.59	64.48	13.38
	≥5	2 (0.7)	14.00	5.66	13.50	6.36	9.00	4.24	7.00	1.41	43.50	17.68
	Test and significance		F = 1.466 p = 0.212		F = 1.070 p = 0.372		F = 0.985 p = 0.416		F = 0.571 p = 0.684		F = 1.055 p = 0.379	
Economic level	Income < expenses	28 (9.2)	18.29	5.29	16.54	5.97	12.82	4.60	9.64	4.05	57.29	18.72
	Income = expenses	248 (81.0)	19.16	5.97	17.79	6.01	13.21	4.97	9.87	3.48	60.02	19.12
	Income > expenses	30 (9.8)	18.27	6.63	16.30	7.18	12.97	5.76	8.93	3.86	56.47	22.32
	Test and significance		F = 0.511 p = 0.600		F = 1.191 p = 0.305		F = 0.098 p = 0.907		F = 0.930 p = 0.396		F = 0.635 p = 0.531	
Job	Housewife	145 (47.4)	19.62	5.91	18.10	6.32	13.70	5.40	10.03	3.73	61.45	20.14
	Civil servant	42 (13.7)	18.00	6.16	17.79	6.28	13.14	5.05	9.98	3.63	58.90	20.26
	Worker	31 (10.1)	8.97	5.49	6.52	5.30	2.42	3.61	9.87	3.28	57.77	5.62
	Self-employed	20 (6.5)	18.20	7.52	15.70	7.12	12.05	5.03	9.05	3.63	55.00	22.43
	Tradesman	17 (5.6)	17.82	5.73	17.35	6.08	12.76	5.09	9.88	4.37	57.82	20.09
	Private sector	35 (11.4)	18.54	4.88	17.09	5.52	12.66	4.75	8.94	3.13	57.23	17.28
	Other	16 (5.2)	19.13	7.38	17.06	5.76	12.44	3.88	9.00	2.16	57.63	17.63
	Test and significance		F = 0.659 p = 0.683		F = 0.702 p = 0.648		F = 0.690 p = 0.658		F = 0.724 p = 0.630		F = 0.591 p = 0.737	
Frequency of using technological devices	Almost never	21 (6.9)	16.90	6.60	14.29	5.94	11.57	4.70	8.38	3.58	51.14	19.13
	Several times a month	5 (1.6)	22.60	5.68	24.20	5.07	19.20	4.97	13.20	2.68	79.20	17.57
	Several times a week	15 (4.9)	17.00	5.04	13.93	5.50	11.60	4.29	8.33	3.29	50.87	16.73
	Almost every day	233 (76.1)	19.24	5.99	17.87	6.10	13.27	5.07	9.91	3.59	60.28	19.51
	Almost always	32 (10.5)	18.94	5.49	17.78	5.42	13.13	4.37	9.66	3.22	59.50	17.26
	Test and significance		F = 1.629 p = 0.167		F = 4.650 p = 0.001		F = 2.808 p = 0.026		F = 2.712 p = 0.030		F = 3.191 p = 0.014	
Competence in using technological tools	Very good	12 (3.9)	19.00	6.02	17.00	6.44	12.00	5.38	9.08	3.42	57.08	20.14
	Good	140 (45.8)	18.53	5.63	17.28	5.91	12.96	4.84	9.61	3.45	58.39	18.61
	Moderate	130 (42.5)	19.36	6.39	17.73	6.44	13.36	5.19	9.92	3.80	60.37	20.42
	Poor	21 (6.9)	20.00	5.54	18.48	5.97	13.76	5.18	9.95	3.38	62.19	19.34
	Incompetent	3 (1.0)	17.33	6.43	15.67	4.16	13.00	2.65	10.67	1.15	56.67	13.05
	Test and significance		F = 0.540 p = 0.706		F = 0.308 p = 0.873		F = 0.341 p = 0.850		F = 0.288 p = 0.885		F = 0.340 p = 0.851	
Playing digital games	Yes	101 (33.0)	19.52	5.61	18.35	5.84	13.75	5.13	10.13	3.51	61.75	18.86
	No	205 (67.0)	18.73	6.13	17.12	6.24	12.85	4.92	9.57	3.59	58.27	19.60
	Test and significance		t = 1.100 p = 0.272		t = 1.648 p = 0.100		t = 1.481 p = 0.140		t = 1.481 p = 0.140		t = 1.479 p = 0.140	
Average time spent playing digital games a day	None	203 (66.3)	18.77	6.15	17.17	6.27	12.87	4.95	9.59	3.61	58.39	19.69
	0–15 minutes	10 (3.3)	17.70	6.04	14.90	4.98	10.60	3.69	8.50	2.72	51.70	15.63
	15–30 minutes	20 (6.5)	19.90	6.33	18.40	7.76	14.50	6.18	10.15	4.15	62.95	22.97
	30 minutes–1 hour	33 (10.8)	19.36	5.86	18.82	5.78	14.06	5.25	10.45	3.46	62.70	19.55
	1 hour	18 (5.9)	19.83	3.47	18.67	3.56	13.50	3.03	10.00	3.18	62.00	11.45
	2 hours	16 (5.2)	20.69	5.61	19.38	5.29	14.19	5.00	11.19	3.37	65.44	18.26
	3 hours	4 (1.3)	17.75	8.06	16.75	6.65	15.00	7.75	8.50	3.42	58.00	25.10
	≥4 hours	2 (0.7)	14.00	2.83	13.00	5.66	11.00	7.07	6.50	0.71	44.50	16.26
	Test and significance		F = 0.647 p = 0.717		F = 1.091 p = 0.369		F = 1.072 p = 0.381		F = 1.150 p = 0.332		F = 0.974 p = 0.450	
Average time the child spends playing digital games a day	None	38 (12.4)	16.53	6.72	14.74	6.42	10.84	4.84	8.42	3.98	50.53	21.04
	0–15 minutes	13 (4.2)	15.92	4.61	16.31	6.34	13.31	5.53	9.00	3.70	54.54	18.34
	15–30 minutes	18 (5.9)	18.78	7.90	16.67	7.59	12.89	6.32	9.06	4.08	57.39	24.72
	30 minutes–1 hour	54 (17.7)	19.76	5.67	18.80	5.79	13.65	5.19	10.50	3.54	62.70	18.92
	1 hour	79 (25.8)	19.22	5.28	17.77	5.34	13.16	3.99	9.97	2.87	60.13	16.16
	2 hours	66 (21.6)	19.85	5.66	18.55	6.06	13.82	5.24	10.12	3.78	62.33	19.58
	3 hours	24 (7.8)	20.08	5.94	17.04	6.24	13.71	5.16	9.88	3.83	60.71	19.91
	≥4 hours	14 (4.6)	18.64	6.95	17.07	7.25	13.50	5.53	8.93	3.34	58.14	21.32
	Test and significance		F = 1.920 p = 0.066		F = 1.932 p = 0.064		F = 1.478 p = 0.175		F = 1.548 p = 0.151		F = 1.792 p = 0.088	

There was no significant relationship between mean scores on the DGASC total and tolerance development during playtime and the value placed on play, excessive focus on playing digital games and conflict, postponement of individual and social duties/tasks, and psychological-physiological reflection of deprivation and immersion in play sub-dimensions and the DPAS total and sub-dimensions (p > 0.05) (Table 5). Since there was no relationship between the total scores of the two scales, no regression analysis was performed.

**Table 5:** Examination of the correlation between DPAS and DGASC – Ankara, Turkey, 2024.

		Approval of effective use of digital media	Protection from digital media risks	DPAS
Tolerance development during playtime and the value placed on play	r	0.011	-0.097	-0.051
	p	0.844	0.089	0.372
Excessive focus on playing digital games and conflict	r	0.023	-0.074	-0.031
	p	0.693	0.197	0.591
Postponement of individual and social duties/tasks	r	0.003	-0.074	-0.042
	p	0.958	0.194	0.461
Psychological-physiological reflection of deprivation and immersion in play	r	0.017	-0.077	-0.055
	p	0.773	0.182	0.342
DGASC	r	0.008	-0.087	-0.046
	p	0.884	0.131	0.418

## DISCUSSION

The investigation of the effect of digital parenting attitudes on digital game addiction is critical to have some insights into the effects of increasing digital game use on students today. Students playing digital games uncontrollably are at risk for digital game addiction^(4)^. According to our research findings, 83% of the students and 33% of their parents who participated in the research stated that they played digital games. More than half of the students played digital games for at least one hour a day, nearly half of them stated that their parents did not know anything about the content of the digital games they played, and 36.3% stated that their parents did not control their game playing. In addition, 59.8% of the parents reported that their children played digital games for at least one hour a day on average. Digital games attract the attention of people of all ages, but the increase in the time student spend playing digital games shows that they are at risk for digital game addiction. Starting to play digital games at an early age, playing games without supervision, and parents playing digital games increase the risk of addiction. Knowing these risks is very critical in terms of taking preventive measures^(2,22)^. According to our research findings, it can be said that students spend a lot of time in front of the screen playing digital games. Similar to our study findings, the number of children playing digital games was reported to be quite high in national and international literature. According to the research conducted by the Turkish Statistical Institute (TURKSTAT), 82.7% of children in the 6–15 age group used the Internet, and 36% of those who used the Internet played digital games. According to this research by TURKSTAT, 94.7% of children in the 6–15 age group who stated that they played digital games played these games regularly, almost every day, or at least once a week^(23)^. According to the “Newzoo 2020 Global Games Market Report”, the number of global digital game players reached 3.2 billion and is expected to reach 3.5 billion in 2025^(24)^. Playing digital games has become almost inevitable today. At this point, considering the risks and possibilities, regulating their use according to the child’s age and needs is among parents’ important responsibilities^(25)^. Nurses can monitor the effects of digital games on children and inform families and students for balanced use. They can raise awareness about play time, physical health and psychological effects, and encourage healthy gaming habits. It is important to ensure that children use digital games safely and educationally by collaborating with families. Nurses can help create policies for digital game use in schools so that children can have fun and continue their healthy development. This approach aims to reduce the negative effects of digital games while supporting their positive aspects. There are several important situations among the nursing diagnoses that school health nurses may encounter regarding digital game use. Lack of physical activity, playing games for long periods of time, inactivity and posture disorders can lead to obesity risk. Eye strain, spending long hours in front of the screen can cause complaints such as pain, burning and blurred vision in the eyes. Sleep disorders, playing games until late at night can disrupt children’s sleep patterns and cause problems such as insomnia and waking up late. Social isolation, excessive digital game use can prevent children’s social interactions, which can lead to social skill deficiencies. Emotional and psychological stress, violent games or failures can lead to emotional problems such as anxiety and depression. Finally, mental overload, is a situation where excessive game playing can cause distraction and a decrease in academic success. Nurses can take these diagnoses into consideration and make interventions^(11,26,27)^. In our research, the mean DGASC score was 59.42. Considering this mean score, students were in the risk group for digital game addiction. According to the digital game addiction evaluations in our research, 14.4% of the students were found to be “addicted,” 2.6% were “highly addicted,” and 54.9% were in the “risky” group. In line with our findings, we can say that digital games are a popular activity in the lives of middle school students, but they create an addiction risk for the students. In a similar study in the literature, it was stated that digital games were a popular activity for students^(28)^. In this study with 1,046 middle school students, they found the mean DGASC score (47.74) to be at a normal level for digital game addiction. They also stated that 7.4% of their participants were “addicted,” 1.5% were “highly addicted,” and 33.9% were in the “at risk” group. According to the Turkish Statistical Institute, the rate of information technology use in children was 50.8% in 2013 and reached 82.7% in 2021, which reveals the likelihood of a critical increase in the rate of addicted individuals that may be encountered in the following years^(23)^. Therefore, the necessary interventions regarding the effects of growing game addiction are of critical importance not only for those who are addicted to games but for all students. Interventions should also focus on preventing game addiction in children who are not already affected by game addiction. Preventive practices should include sharing information about game addiction, the potential dangers of excessive exposure to technology, and content-appropriate games, and limiting screen time to less than one hour a day. Educational programs to prevent digital game addiction should be supported, and policies should be developed to limit personal electronic device use during formal education. For children with game addiction, methods such as cognitive behavioral therapy and family therapy should also be used in the treatment of digital game addiction^(10)^. In a school where students are at risk of digital game addiction, nurses can take various precautions. First, they can raise awareness by educating students about the harms of game addiction. They can offer suggestions for limits and balanced use of screen time. They can also help families develop healthy habits at home by providing guidance on game duration and content. Nurses can organize group activities and encourage physical activity for students to develop social interaction skills. They can conduct individual interviews with students who show signs of addiction and provide referrals where they can receive professional support when necessary. This approach encourages healthy game habits^(26,27)^.

In our study, the difference between the mean DGASC total score according to the variable of skipping school to play digital games was found to be statistically significant. The mean scores of students who did not skip school were higher. When the literature was examined, it was seen that digital game addiction caused academic failure^(29,30)^. Contrary to the literature, the reason why the mean scores of students who did not skip school on the digital game addiction scale were significantly higher in our findings may have been due to the students’ interest in digital games. The interest of students who did not skip school in digital games may have developed more as a result of their interactions with their friends. In addition, students who went to school regularly may have learned to manage their time better, but they may have spent most of their free time after school playing digital games. Another reason may have been that the difficulties brought by school life caused students to prefer digital games as a means of escape. Nurses can take various initiatives against students’ digital game addiction. First, they can raise awareness by informing students and families about the effects of games on academic success. They can also provide guidance on developing time management skills and help students balance their after-school activities. Nurses can organize alternative fun and relaxing activities to support students in coping with stress. They can conduct individual interviews with students who use digital games as an escape tool and direct them to professional support. These initiatives can help students develop healthy habits. Inactivity, nutritional disorders, obesity, mental disorders, and many other problems arise due to digital game addiction. Adolescents addicted to digital games do not exercise, and the increased time spent in front of the game leads to many problems, such as musculoskeletal disorders, obesity, eye diseases, and nutritional disorders. Playing games for a long time can also trigger disruptions in social life, withdrawal from friends, and an asocial lifestyle. As the time spent playing games increases, the number of homework forgetting increases, and the level of academic success decreases^(12,13)^.

The scores of eighth-grade students from the “postponement of individual and social duties/tasks” sub-dimension, which is one of the sub-factors of the digital game addiction scale, were found to be significantly higher. Similarly, in the literature, studies on the examination of the relationship between digital game addiction of middle school students and their demographic data indicated that eighth-grade students had higher digital game addiction levels than those in other grades^(31,32)^. Another study conducted by Gül and Özgür showed a significant difference between students’ digital game addiction levels and their grade levels^(28)^. It was emphasized in the literature that screen use increased as the child got older and that the vast majority of children had screen time well above the recommended limits by the American Pediatric Association^(25)^. Reasons why students’ addiction levels increase as the grade level increases were that they may have become more independent from their families, become autonomous, and socialized with the increase in their age and the effect of puberty, and that they may have turned more to virtual environments and therefore to digital games, which are a popular activity for children, due to their desire to be accepted in a group. In addition, the reason why 8^th^-grade students had higher digital game addiction levels than 5th, 6th, and, 7th- grade students may have been because students in this age group are more familiar with technology and play more digital games with their friends due to their developing social circles.

The difference between the mean DGASC total scores according to parents’ gender was statistically significant. The mean scores of the children of female parents were significantly higher. The examination of parents’ demographic data in our study indicated that the majority were mothers (67.6%). Our findings showed that the children of parents who were mothers had higher digital game addiction scores than the scores of the children whose parents were fathers. Doğan and Döğer found a positive relationship between the time spent on the Internet daily by the mothers and their children^(19)^. Considering that mothers usually spend more time communicating with their children as parents than fathers, the time mothers spend on technological devices will be free time for the child. It is an expected result that the child spends time on technological devices as a result of taking the mother as a role model during this time. The time mothers who prioritize taking care of their children spend on the Internet affects the children^(19,33)^. Nurses can develop intervention strategies by considering parents’ digital media use and its effects on children. Nurses can organize awareness programs aimed at preventing mothers from increasing their use of technology and provide mothers with training on digital addiction. They can also guide families so that children can use technology in a healthy way by strengthening communication with parents. Nurses can encourage mothers to spend more quality time with their children and balance technology with social interaction and physical activities. In this way, they can help children gain healthy habits by reducing their risk of digital game addiction^(11)^.

The difference between the mean DGASC total scores was statistically significant according to how often parents used technological devices. In the advanced analysis conducted to determine which group caused the difference according to the frequency of use of technological devices, it was found that the mean scale scores of children whose parents used these devices several times a month were higher than the scores of other groups. In a study on the relationship between parents and children in terms of internet addiction, Doğan and Döğer concluded that there was a significant relationship between the time spent on technological devices daily by parents and their children^(19)^. Parents are role models for their children in technology use. When parents communicate face-to-face with their children less, their children may tend to turn to digital games more. As a result of this situation, digital game addiction may increase. Since parents are the closest role models for their children, the actions taken by them have a great effect on children. Children whose parents play digital games are at greater risk of becoming addicted to these games^(28)^. It is important for nurses to develop various strategies for parents’ technology use habits. Nurses can provide education to parents about the effects of digital media use on children and provide guidance on ways to balance children’s interest in technology. They can also conduct awareness-raising activities on parents limiting technology use and engaging in more face-to-face communication. They can provide guidance emphasizing that parents should balance the time they use digital media, without forgetting that they are role models for their children. By raising awareness in families on this issue, nurses can help children gain healthy habits to prevent digital game addiction^(11)^.

In the study, the mean score on the total Digital Parenting Attitude Scale was 46.97. The rapid increase in the use of digital technologies by children draws attention to the importance of parents’ attitudes toward these technologies. There is a need to raise parental awareness so that children can benefit from the learning opportunities offered through digital technologies and be protected from their risks^(4)^. It is important to evaluate parents’ attitudes so that children can benefit from the opportunities of technology through their parents and be protected from its risks. This is because attitudes are cognitive and affective frameworks and are considered permanent and consistent determinants in predicting behavior^(4)^. For this reason, parents should be able to use appropriate digital parenting roles in creating and managing safe environments suitable for their children’s learning needs and they should be able to exhibit attitudes that are effective in choosing these roles. The high-level digital parenting attitudes of today’s parents have become very important in terms of raising their awareness of the benefits and risks of technology, taking the necessary precautions in this regard, and being able to guide their children. Parental attitudes have a great impact on children’s development, both socially and cognitively. At this point, parental attitudes should also be supportive^(34)^. Nurses can organize educational programs that explain to parents the effects, benefits, and potential risks of digital technologies on children. Organizing periodic parent training to improve adolescents’ health behaviors and gain healthy gaming behaviors is also important^(12)^. It can be emphasized that parents need to develop a conscious and supportive attitude to ensure that their children are safe in the digital world. They can also create awareness about digital parenting and provide guidance to parents so that their children can establish healthy relationships with digital games and technologies. Knowing the factors affecting adolescents’ game addiction is very important for pediatric nurses to identify adolescents who are at risk for game addiction and take necessary precautions^(12,26,27)^.

In the study, the relationship between the Digital Parenting Attitude Scale and the Digital Game Addiction Scale for Children was examined and no significant relationship was found. Contrary to our findings, some studies in the literature have shown that parental attitudes are related to children’s game addiction levels^(19,25,35,36,37)^. The reason why we did not find a significant relationship between digital parenting attitudes and digital game addiction of students in this study may have been that the parents in our study group had not been informed about the potential harms of digital games. According to our study findings, almost all of the students and 33% of the parents stated that they played digital games. In addition, more than half of the students played digital games for at least one hour a day, almost half of the students reported that their parents did not know anything about the content of the digital games they played, and 36.3% stated that their parents did not control them while playing digital games. All these findings may suggest that parents had low awareness about and inadequate control over their children’s digital game playing, did not guide their children in digital games, and ignored the possible risks in the digital world. By supporting parents’ attitudes towards digital media use, nurses can help children develop healthy habits that will keep them safe in the digital world. Nurses can assess students’ gaming behaviors in both clinical practice and research and leverage online digital intervention programs aimed at enhancing coping strategies and skills^(27)^.

## CONCLUSION

In this study, in which we investigated the effects of digital parenting attitudes on digital game addiction in middle school students, it was found that approximately three-quarters of the students played digital games and that more than half played these games for at least one hour a day. When the students were evaluated according to their digital game addiction levels, it was determined that approximately three-quarters were game addicts or in the risk group. No significant relationship was found between digital parenting attitudes and students’ digital game addiction levels.

Nurses can provide support for parents and students to reduce the risk of digital game addiction in children. It is very important to organize digital game addiction training programs for parents and to emphasize that they should be positive role models for their children in these programs. Nurses can guide parents in setting limits on their children’s digital game playing, raising awareness about safe online behaviors, and monitoring their children’s gaming habits. In addition, they can increase students’ awareness of this issue by organizing programs about digital game addiction. Digital game addiction screenings should also be included in the periodic health screening programs conducted by the nurse. Nurses can support students who are digital game addicts and at risk by holding individual interviews and can ensure that they receive professional help when necessary. They can teach students preventive strategies such as time management and stress coping. It is very important to add protective and preventive lessons to the education curriculum to prevent digital game addiction. Digital game addiction is common among students. According to our findings, approximately three-quarters of students are game addicts or in the risk group. Nurses should develop strategies to prevent digital game addiction. Digital parenting attitudes should be taken into consideration when examining digital game addiction in students. Nurses should organize training programs for students and parents to prevent digital game addiction.

## IMPLICATIONS FOR NURSING PRACTICE

Nurses should detect digital game addiction early and be ready for intervention, and they should be able to evaluate its risks and symptoms. They should use valid and reliable scales to detect digital game addiction in students. Nurses should plan training programs for teachers, students, and parents on digital game addiction. School-based intervention programs can be created to prevent and reduce digital game addiction. Students who are addicted to digital games and at risk of game addiction should receive professional help. In addition, our findings will guide nurses in planning interventions. Our research revealed that approximately three-quarters of the students were addicted to games or were in the risk group. Nurses should consider this situation and make plans to reduce the level of game addiction by collaborating with students, parents, and psychological counseling and guidance teachers at schools. In this context, awareness projects that include teachers, students, and parents should be planned.

## STRENGTHS AND LIMITATIONS

The data of this study were collected from both parents and students to reveal the effect of digital parenting attitudes on digital game addiction in middle school students. This situation constituted the strength of the study as it increased the reliability of the data collected. The data were limited to the responses of 306 students from a state middle school and 306 parents in the XX. Therefore, the results obtained in the study can only be generalized to this group.

## DATA AVAILABILITY

The data are available from the corresponding author on reasonable request.
