# Supplementary material for: Examining the effect of digital parenting attitudes on digital game addiction
Source: Rev Esc Enferm USP. 2025 Jul 28;59:e20250059. doi: 10.1590/1980-220X-REEUSP-2025-0059en (PMC12309521; doi:10.1590/1980-220X-REEUSP-2025-0059en)
Supplement: Translation

# Translation: Examen del efecto de las actitudes parentales digitales en la adicción a los juegos digitales

**DOI:** 10.1590/1980-220X-REEUSP-2025-0059es

**Authors:** Handan Kabadayı, Aslıhan Öztürk Eyimaya

**Affiliations:** 1 Ankara University, Graduate Schools of Health Sciences, Ankara, Turkey.; 2 Ankara University, Faculty of Nursing, Ankara, Turkey.

**Keywords:** Adicción, Digital, Adicción a los juegos, Enfermeros, Crianza, Estudiante

## Abstract

**Objetivo:**

Este estudio se llevó a cabo para investigar el efecto de las actitudes de crianza digital en la adicción a los juegos digitales en estudiantes de secundaria. Las enfermeras pueden brindar apoyo a los padres y estudiantes para reducir el riesgo de adicción a los juegos digitales en los niños.

**Métodos:**

Se utilizó un diseño transversal en este estudio. La investigación se realizó en una escuela secundaria en X. La muestra del estudio consistió en 306 estudiantes y 306 padres. Se utilizaron un “Formulario de Información Personal”, la “Escala de Actitud de Crianza Digital” y la “Escala de Adicción a Juegos Digitales para Niños” para recolectar los datos.

**Resultados:**

La tasa de juego digital fue del 83% entre los estudiantes, y el 24,8% jugaba juegos digitales un promedio de una hora al día. La puntuación media de los padres en la “Escala de Actitud de Crianza Digital” fue de 46,97 ± 6,27. La puntuación media de los estudiantes en la “Escala de Adicción a Juegos Digitales” fue de 59,42 ± 19,39. Al evaluar a los estudiantes según sus niveles de adicción a los juegos digitales, se determinó que el 14,4% eran “adictos”, el 2,6% “altamente adictos” y el 54,9% estaban en el grupo “en riesgo”. No se encontró una relación significativa entre las escalas (p > 0,05).

**Conclusión:**

No se encontró una correlación significativa entre las escalas. Sin embargo, se observó que la mayoría de los estudiantes eran adictos a los juegos digitales o estaban en el grupo de riesgo. Se recomienda que se planifiquen las intervenciones necesarias en cooperación con los padres, para que los estudiantes puedan mantener comportamientos saludables en el juego digital y reciban formación preventiva y de protección para evitar la adicción a los juegos digitales.

## INTRODUCCIÓN

Los juegos digitales se han convertido en una de las fuentes de entretenimiento y actividad más populares entre los estudiantes de hoy en día. Los hábitos de juego excesivos e incontrolados pueden convertirse en adicción a los juegos digitales. La adicción a los juegos digitales puede tener efectos negativos en la salud física, social y emocional de los estudiantes y repercutir en su vida cotidiana^(1,2)^. Por estas razones, las actitudes adoptadas por los padres para equilibrar y controlar el uso de los juegos digitales tienen un papel importante a la hora de determinar el riesgo de adicción a los juegos en sus hijos. En la adicción a los juegos digitales, jugar se convierte en un objetivo para el individuo. El individuo se aísla del mundo real, desarrolla tolerancia a los juegos y muestra síntomas de abstinencia cuando no juega, al igual que en otros tipos de adicción^(3)^.

La necesidad de prevenir la adicción a los juegos digitales y de concienciar a los padres sobre el uso correcto de la tecnología ha llevado a la aparición del concepto de «paternidad digital»^(1)^. El objetivo de la crianza digital es ayudar a los niños a utilizar correctamente las tecnologías digitales, protegiéndolos de los riesgos en línea y ayudándoles a desarrollar sus habilidades sociales, emocionales y cognitivas en el mundo digital. En este contexto, los padres informan y orientan a sus hijos sobre el uso de la tecnología digital y los comportamientos en línea^(3)^. Las actitudes parentales digitales incluyen las percepciones, los enfoques y los comportamientos de los padres sobre cómo utilizarán sus hijos las tecnologías digitales. Las actitudes de los padres hacia el uso de juegos digitales por parte de sus hijos pueden afectar a sus hábitos de juego y reducir o aumentar el riesgo de adicción a los juegos digitales^(4)^. La supervisión y orientación de los padres sobre el uso de los juegos digitales por parte de sus hijos desempeña un papel importante en la reducción del riesgo de adicción a los juegos digitales. Cuando los padres establecen normas coherentes para la selección de juegos y determinan su duración, ayudan a los niños a regular sus hábitos de juego. Además, establecer normas coherentes anima a los niños a compartir sus preocupaciones y problemas sobre los juegos con sus padres y ayuda a detectar a tiempo posibles síntomas de adicción. Las actitudes de los padres en relación con los juegos digitales influyen en el uso que hacen los niños de los juegos digitales y, por tanto, determinan el riesgo de adicción a los juegos digitales^(4,5)^. Algunos estudios realizados para revelar la relación entre las actitudes de los padres en relación con los juegos digitales y la adicción a los juegos digitales han demostrado que las actitudes de los padres pueden influir en los hábitos de juego de los niños^(3,4)^. Además, en la literatura se ha afirmado que el tiempo que los niños dedican a las herramientas digitales es directamente proporcional al tiempo que dedican los padres^(6)^.

Para prevenir la adicción a los juegos digitales, los niños deben recibir formación desde una edad temprana sobre el uso correcto de las tecnologías digitales, los riesgos y la determinación del tiempo y el contenido adecuados. Los enfermeros pueden ofrecer apoyo a padres y alumnos para reducir el riesgo de adicción a los juegos digitales en los niños. Pueden ofrecer formación y asesoramiento a los padres sobre la crianza digital. Pueden orientar a los padres sobre cómo poner límites a los juegos digitales de sus hijos, concienciarlos sobre comportamientos seguros en línea y monitorear los hábitos de juego de sus hijos^(2,7)^. Con la educación y el asesoramiento que proporcionan los enfermeros, los padres pueden desarrollar estrategias más eficaces para equilibrar el uso de juegos digitales de sus hijos y reducir el riesgo de adicción. Además, pueden educar a los alumnos sobre los efectos de los juegos digitales. Pueden aumentar la concienciación de los estudiantes sobre este problema organizando programas de sensibilización sobre la adicción a los juegos digitales^(2,8,9)^. Pueden apoyar a los estudiantes que muestren signos de adicción a los juegos digitales manteniendo entrevistas individuales con ellos y asegurándose de que reciben ayuda profesional cuando sea necesario. También pueden enseñar a los estudiantes estrategias preventivas, como la gestión del tiempo y las habilidades para afrontar el estrés. Con la educación, el apoyo y la orientación que proporcionan a padres y alumnos, las enfermeras pueden ayudar a los niños a desarrollar hábitos saludables de uso de la tecnología digital y reducir el riesgo de adicción a los juegos digitales. Las enfermeras deben asumir un papel activo en la adicción a los juegos digitales y apoyar los hábitos de uso de la tecnología digital de los niños^(8,10)^. La adicción a los juegos digitales es común entre los estudiantes. La adicción a los juegos digitales puede causar problemas como el deterioro de la interacción social y la comunicación. Las enfermeras pueden evaluar los comportamientos de juego de los estudiantes. Las enfermeras deben desarrollar estrategias para prevenir la adicción a los juegos digitales^(11)^. Las actitudes digitales de los padres deben tenerse en cuenta al examinar la adicción a los juegos digitales en los estudiantes. Las enfermeras deben organizar programas de formación para prevenir la adicción a los juegos digitales. Las actitudes digitales de los padres desempeñan un papel importante en la configuración de las relaciones de los niños con los juegos digitales. En este contexto, los enfoques positivos o negativos de los padres hacia el uso de los medios digitales pueden afectar directamente al riesgo de los niños de desarrollar adicción a los juegos. Los bajos niveles de conciencia digital de los padres pueden hacer que los niños pasen tiempo sin control frente a la pantalla. Las actitudes digitales excesivamente controladoras o completamente desinteresadas también pueden desencadenar comportamientos adictivos en los niños. Esto puede afectar negativamente al desarrollo psicosocial, el éxito académico y la salud física de los niños. Las enfermeras de programas escolares desempeñan un papel fundamental a la hora de monitorear y orientar los hábitos de juego digital de los niños. Las enfermeras pueden ayudar a los niños a adquirir hábitos digitales saludables informando a los padres sobre sus actitudes digitales. Pueden organizar programas educativos sobre el uso de los medios digitales en colaboración con las familias y concienciar sobre los signos tempranos y los factores de riesgo relacionados con la adicción a los juegos. También pueden ofrecer asesoramiento individual a los alumnos con riesgo de adicción a los juegos y remitirlos a los profesionales sanitarios pertinentes cuando sea necesario. En este contexto, es de gran importancia que las enfermeras sanitarias escolares asuman un papel tanto preventivo como de apoyo en la lucha contra la adicción digital^(11–13)^.

Existen algunos estudios en la literatura sobre la adicción a los juegos digitales y las actitudes de los padres^(6,14,15)^. Sin embargo, no existen estudios sobre el impacto de las actitudes parentales digitales en la adicción a los juegos digitales en estudiantes de secundaria. Este estudio es original en el sentido de que se llevó a cabo para investigar el efecto de las actitudes parentales digitales en la adicción a los juegos digitales en estudiantes de secundaria. Por lo tanto, se cree que los resultados de la investigación contribuirán a la literatura.

Las preguntas de investigación son las siguientes

1. ¿Cuál es el nivel de adicción a los juegos digitales de los alumnos?
2. ¿Cuál es el nivel de actitud de los padres hacia los juegos digitales?
3. ¿Existe una diferencia significativa entre las características descriptivas de los alumnos y las puntuaciones medias de la «Escala de adicción a los juegos digitales para niños»?
4. ¿Existe una diferencia significativa entre las características descriptivas de los padres y las puntuaciones medias de la «Escala de adicción a los juegos digitales para niños»?
5. ¿Existe una correlación significativa entre las puntuaciones medias de la «Escala de actitudes digitales de los padres» (DPAS) y la «Escala de adicción a los juegos digitales para niños» (DGASC)?

## MÉTODO

### Diseño y Participantes

Se empleó un diseño transversal. Esta investigación se llevó a cabo para investigar el efecto de las actitudes de crianza digital en la adicción a los juegos digitales en estudiantes de secundaria que asisten a una escuela secundaria ubicada en X. Los datos de la investigación se recolectaron entre diciembre de 2023 y febrero de 2024. De acuerdo con la Investigación del Ranking de Desarrollo Socioeconómico 2022, cuyo objetivo era medir el nivel de desarrollo socioeconómico de los distritos de nuestro país por distritos, el distrito de Mamak tiene un nivel de desarrollo de segundo grado. Es uno de los centros receptores de migración de otros asentamientos. Los distritos de este nivel proporcionan empleo en la industria manufacturera y en el sector servicios. La infraestructura básica de estos distritos está casi terminada, y la calidad de la educación, la sanidad y la vida es alta en comparación con muchos distritos^(16)^. Dado que todas las escuelas públicas de nuestro país reciben la misma financiación del gobierno, la escuela del distrito de Mamak donde se realizaría el estudio se seleccionó por sorteo, sin aplicar un procedimiento de clasificación socioeconómica.

Se encontraban matriculados 1.511 alumnos en la escuela en las fechas en que se recolectaron los datos. Así pues, la población del estudio estaba formada por 1.511 alumnos de secundaria y 1.511 padres. Se empleó la fórmula de muestreo de la población conocida para determinar el tamaño de la muestra del estudio^(17)^. Según la fórmula, se incluyeron en la muestra al menos 306 alumnos y 306 padres. El número de alumnos de cada rama que debía incluirse en la muestra se determinó calculando su proporción con respecto a la población general. Los alumnos se dividieron en cuatro estratos según sus cursos (5º, 6º, 7º y 8º). El número de alumnos que debían incluirse en la muestra se determinó con una selección proporcional de cada estrato según el número de alumnos de los estratos, y los alumnos de cada estrato se seleccionaron por el método de muestreo aleatorio simple. Todos los alumnos de las ramas se enumeraron y numeraron mediante listas de clase. A continuación, se determinaron los alumnos que debían incluirse en la muestra mediante una tabla de números aleatorios. El proceso de selección continuó hasta que se alcanzó el tamaño de muestra determinado de cada rama. La muestra incluía 83 alumnos de quinto curso, 74 de sexto, 79 de séptimo y 70 de octavo.

Los criterios de inclusión eran tener entre 10 y 14 años, ser padres de alumnos de esa edad y aceptar voluntariamente participar en el estudio. Los criterios de exclusión fueron discapacidad visual o auditiva o una enfermedad neuropsiquiátrica en el estudiante o en los padres, o entregar un formulario de recolección de datos incompleto.

### Instrumentos

Los instrumentos para recolectar datos incluyeron un formulario de información personal para estudiantes y padres, la Escala de Actitud de Padres Digitales y la Escala de Adicción a Juegos Digitales para Niños.

### Formulario de Información Personal

Este formulario fue preparado por los investigadores tras una revisión de la literatura. Incluía preguntas sobre la edad, el sexo, el curso, la afición a los juegos digitales de los alumnos y la afición a los juegos digitales de sus padres, así como sobre la edad, el sexo, el nivel educativo, la ocupación, el nivel socioeconómico y el tiempo diario de uso de dispositivos tecnológicos por parte de los padres^(18,19)^.

### Escala de Actitudes Digitales de los Padres (DPAS)

Esta herramienta fue desarrollada por İnan Kaya et al.^(4)^ para determinar las actitudes de los padres hacia el uso de herramientas de medios digitales por parte de sus hijos. Consta de 12 ítems utilizados para medir las actitudes parentales digitales de los padres con hijos de entre 6 y 18 años. La escala consta de dos subdimensiones: «aprobación del uso efectivo de los medios digitales» y “protección frente a los riesgos de los medios digitales”. Para evaluar los ítems de la escala se utiliza una escala tipo Likert de cinco puntos. Las puntuaciones de la escala oscilan entre 12 y 60. El coeficiente alfa de Cronbach de las subdimensiones es de 0,77 y 0,72, respectivamente. Los coeficientes alfa de las subdimensiones fueron de 0,82 y 0,86, respectivamente. El valor alfa de la DPAS total se estimó en 0,87.

### Escala de Adicción a Los Juegos Digitales Para Niños (DGASC)

Esta escala fue desarrollada por Hazar y Hazar para determinar el nivel de adicción a los juegos digitales en niños de entre 10 y 14 años^(20)^. Consta de 24 ítems y cuatro subdimensiones: «desarrollo de la tolerancia durante el tiempo de juego y el valor otorgado al juego», “excesiva concentración en jugar a juegos digitales y conflicto”, “postergación de deberes/tareas individuales y sociales”, “reflejo psicológico-fisiológico de la privación e inmersión en el juego”. Para evaluar los ítems de la escala se utilizó una escala tipo Likert de cinco puntos (1 = en desacuerdo, 2 = en desacuerdo, 3 = indeciso, 4 = de acuerdo, 5 = totalmente de acuerdo). La puntuación más baja de la escala es 24 y la más alta 120. Las puntuaciones se interpretan de la siguiente manera: 1–24: grupo normal; 25–48: grupo de bajo riesgo; 49–72: grupo de riesgo; 73–96: grupo adicto; 97–120: grupo muy adicto. Las puntuaciones altas en la escala indican un alto nivel de adicción a los juegos digitales. El coeficiente alfa de Cronbach es de ,78 para el primer subfactor, ,81 para el segundo subfactor, ,76 para el tercer subfactor, ,67 para el cuarto subfactor y ,90 para la escala total. En este estudio de tesis, el coeficiente alfa de Cronbach resultó ser 0,96 para la escala total y 0,90, 0,88, 0,90 y 0,80 para los subfactores, respectivamente.

### Recolección de Datos

Se informó a la dirección de la escuela sobre la investigación, y se determinaron con los profesores de orientación y asesoramiento psicológico las clases en las que se llevaría a cabo la aplicación y las fechas. Se envió a los padres de los alumnos que participarían en el estudio un formulario de consentimiento informado en sobre cerrado, que los profesores recogieron de los alumnos en el plazo de una semana. Se visitaron las clases en compañía de los profesores de orientación y asesoramiento psicológico, se informó a los alumnos sobre el estudio y se obtuvo el consentimiento verbal de los que aceptaron participar en él. Se distribuyeron a los alumnos instrumentos para recolectar datos y se les pidió que los rellenaran bajo la supervisión del investigador. Los cuestionarios que rellenarían los padres que aceptaron participar en el estudio se entregaron a los alumnos para que se los llevaran a sus padres, y se recolectaron los datos. Se tardó aproximadamente 15 minutos en rellenar las escalas.

### Consideraciones Éticas

Para la realización del estudio se obtuvo la aprobación del XX Comité de Ética (fecha: 20/06/2023 y número: 11/88) y el permiso institucional del XX (fecha: 29/09/2023 y número: 1093612). Se obtuvo el consentimiento verbal de los alumnos y el consentimiento escrito de los padres incluidos en el estudio.

### Análisis de los Datos

Los datos se analizaron en el paquete informático SPSS para Windows 22. En el análisis de los datos se utilizaron cifras, porcentajes, valores mínimos y máximos, media y desviación estándar. El supuesto de normalidad de los datos se calculó con los coeficientes «Kurtosis» y «Skewness» (asimetría) (±2)^(21)^. Se comprobó que todos los datos presentaban una distribución normal. En la comparación de grupos emparejados se utilizó la prueba t de grupos independientes y el análisis de varianza (LSD o prueba de la diferencia mínima significativa, en los casos en que las varianzas eran homogéneas, como análisis avanzado, y Dunnet C en los casos en que no lo eran). Se utilizó el análisis de correlación de Pearson para las inferencias relacionales.

## RESULTADOS

Las puntuaciones medias de los padres fueron 22,88 ± 3,54 en la sub-dimensión de aprobación del uso efectivo de los medios digitales, 24,09 ± 3,70 en la sub-dimensión de protección frente a los riesgos de los medios digitales, y 46,97 ± 6,27 en el total de la Escala de actitud digital parental – DPAS (Tabla 1).

**Tabla 1:** Distribución de las puntuaciones de la Escala de Actitudes Digitales de los Padres y sus subdimensiones (n = 306) – Ankara, Turquía, 2024.

Escalas y sub-escalas	Min.	Max.	Media	DE
Aprobación del uso eficaz de los medios digitales	10,00	30,00	22,88	3,54
Protección frente a los riesgos de los medios digitales	12,00	30,00	24,09	3,70
Escala de actitud digital parental (Digital Parenting Attitude Scale – DPAS)	22,00	60,00	46,97	6,27

Las puntuaciones medias de los niños en la Escala de Adicción a los Juegos Digitales fueron las siguientes: 18,99 ± 5,97 en las subdimensiones de desarrollo de la tolerancia durante el tiempo de juego y valor otorgado al juego; 17,53 ± 6,13 en la subdimensión de excesiva concentración en los juegos digitales y conflicto; 13,15 ± 5,00 en la subdimensión de aplazamiento de deberes/tareas individuales y sociales; 9,75 ± 3,57 en la subdimensión de reflejo psicológico-fisiológico de privación e inmersión en el juego; y 59,42 ± 19,39 en la escala total. Según los puntos de corte de la escala, el 14,4% de los alumnos eran adictos, el 2,6% eran muy adictos y el 54,9% pertenecían al grupo de riesgo (Tabla 2).

**Tabla 2:** Distribución de las puntuaciones obtenidas en la Escala de Adicción a los Juegos Digitales para Niños y sus subdimensiones –Ankara, Turquía, 2024.

Escalas y sub-escalas	n	Min.	Max.	Media	DE
Desarrollo de la tolerancia durante el recreo y valor del juego	306	7,00	35,00	18,99	5,97
Concentración excesiva en los juegos digitales y los conflictos	306	7,00	35,00	17,53	6,13
Aplazamiento de deberes/tareas individuales y sociales	306	6,00	30,00	13,15	5,00
Reflejo psicológico-fisiológico de la privación y la inmersión en el juego	306	4,00	20,00	9,75	3,57
Escala de adicción a los juegos digitales para niños (DGASC)	306	24,00	120,00	59,42	19,39
Distribución de los puntos de corte	n	%	–	–	–
Normal	12	3,9	–	–	–
Bajo riesgo	74	24,2	–	–	–
Riesgoso	168	54,9	–	–	–
Adicto	44	14,4	–	–	–
Altamente adicto	8	2,6	–	–	–

DE: Desvío estándar.

Se observó que la diferencia entre las puntuaciones medias de los alumnos en las subdimensiones «desarrollo de la tolerancia durante el tiempo de juego y valor otorgado al juego» y «reflejo psicológico-fisiológico de la privación e inmersión en el juego» y el DGASC total era estadísticamente significativa según el estado de absentismo escolar para jugar a juegos digitales. Las puntuaciones medias de los alumnos que no faltaron a clase fueron más altas (p < 0,05). La diferencia entre las puntuaciones medias de las subdimensiones de aplazamiento de deberes/tareas individuales y sociales fue estadísticamente significativa según las calificaciones de los alumnos (p < 0,05). Las puntuaciones medias de los alumnos de 8º curso fueron superiores a las de los alumnos de 6º curso (Tabla 3).

**Tabla 3:** Comparación de las puntuaciones de los estudiantes en el DGASC y sus subdimensiones según sus características descriptivas – Ankara, Turquía, 2024.

Características sociodemográficas		n (%)	Desarrollo de la tolerancia durante el recreo y valor del juego		Concentración excesiva en los juegos digitales y los conflictos		Aplazamiento de deberes/tareas individuales y sociales		Reflejo psicológico-fisiológico de la privación y la inmersión en el juego		DGASC	
			Media	DE	Media	DE	Media	DE	Media	DE	Media	DE
Edad	10	41 (13,4)	18,73	5,66	17,98	5,58	12,66	4,59	9,83	3,31	59,20	18,00
	11	61 (19,9)	18,28	5,06	16,87	5,42	11,97	3,77	9,26	3,29	56,38	16,36
	12	89 (29,1)	18,81	6,81	17,38	6,92	13,33	5,56	9,62	3,69	59,13	21,66
	13	76 (24,9)	19,24	5,72	17,83	6,08	13,58	5,23	9,95	3,80	60,59	19,55
	14	39 (12,7)	20,31	6,06	17,82	6,10	14,28	5,12	10,38	3,57	62,79	19,63
	Test y significancia		F = 0,762 p = 0,551		F = 0,309 p = 0,872		F = 1,631 p = 0,166		F = 0,683 p = 0,604		F = 0,744 p = 0,563	
Género	Masculino	113 (36,9)	19,37	5,97	17,91	6,19	13,04	5,17	9,79	3,70	60,12	19,48
	Femenino	193 (63,1)	18,77	5,97	17,30	6,09	13,21	4,91	9,74	3,50	59,02	19,38
	Test y significancia		t = 0,855 p = 0,393		t = 0,841 p = 0,401		t = -0,283 p = 0,777		t = 0,122 p = 0,903		t = 0,478 p = 0,633	
Año escolar	5	83 (27,1)	18,83	5,75	17,80	5,95	12,54	4,62	9,75	3,50	58,92	18,80
	6	74 (24,2)	18,01	5,76	16,55	6,16	12,24	4,66	9,11	3,49	55,92	18,62
	7	79 (25,8)	19,11	6,37	17,66	6,20	13,37	5,19	9,75	3,59	59,89	19,98
	8	70 (22,9)	20,07	5,90	18,09	6,23	14,59	5,33	10,46	3,67	63,20	19,91
	Test y significancia		F = 1,465 p = 0,224		F = 0,880 p = 0,452		F = 3,261 p = 0,022		F = 1,725 p = 0,162		F = 1,737 p = 0,159	
Tener computador/tablet	Si	113 (36,9)	18,69	5,66	17,36	5,71	13,04	4,83	9,35	3,20	58,44	18,04
	No	193 (63,1)	19,17	6,15	17,62	6,37	13,22	5,11	9,99	3,76	59,99	20,17
	Test y significancia		t = −0,672 p = 0,502		t = −0,356 p = 0,722		t = −0,307 p = 0,759		t = −1,571 p = 0,117		t = −0,675 p = 0,500	
Tener un teléfono celular	Si	82 (26,8)	17,99	5,89	16,78	5,87	12,94	4,75	9,10	3,35	56,80	18,57
	No	224 (73,2)	19,36	5,97	17,80	6,21	13,23	5,10	10,00	3,63	60,38	19,64
	Test y significancia		t = −1,784 p = 0,075		t = −1,289 p = 0,198		t = −0,446 p = 0,656		t = −1,958 p = 0,051		t = −1,430 p = 0,154	
Tener internet en casa	Si	276 (90,2)	18,91	5,74	17,41	5,99	13,11	4,89	9,71	3,52	59,14	18,81
	No	30 (9,8)	19,70	7,81	18,63	7,28	13,50	6,01	10,17	4,06	62,00	24,32
			t = −0,536 p = 0,595		t = −1,042 p = 0,298		t = −0,403 p = 0,688		t = −0,665 p = 0,507		t = −0,766 p = 0,444	
Estatus de jugar juegos digitales	Si	254 (83,0)	18,91	5,83	17,37	6,00	13,04	4,89	9,65	3,47	58,96	18,81
	No	52 (17,0)	19,37	6,66	18,31	6,71	13,71	5,54	10,27	4,04	61,65	22,09
	Test y significancia		t = −0,497 p = 0,620		t = −1,009 p = 0,314		t = −0,888 p = 0,375		t = −1,141 p = 0,255		t = −0,911 p = 0,363	
El dispositivo en el que más se juega	Computador/tablet	174 (56,9)	18,89	5,52	17,41	5,77	13,02	4,77	9,76	3,44	59,09	18,19
	Teléfono celular	86 (28,1)	19,34	6,61	17,94	6,66	13,38	5,42	9,69	3,70	60,35	21,02
	Consola de juegos	8 (2,6)	17,88	3,83	16,00	4,69	11,88	2,47	9,75	3,15	55,50	13,14
	Ninguno	38 (12,4)	18,92	6,86	17,42	6,85	13,47	5,54	9,87	4,04	59,68	22,34
	Test y significancia		F = 0,208 p = 0,891		F = 0,318 p = 0,812		F = 0,324 p = 0,808		F = 0,024 p = 0,995		F = 0,193 p = 0,901	
Daily digital game playing time	Ninguno	31 (10,1)	19,32	6,98	17,84	6,95	13,58	5,45	10,23	4,03	60,97	22,51
	0–15 minutos	17 (5,6)	19,59	6,96	18,29	7,46	13,82	6,76	9,59	4,57	61,29	25,24
	15–30 minutos	25 (8,2)	17,12	7,19	16,04	7,13	11,76	5,74	8,92	3,48	53,84	22,15
	30 minutos– 1 hour	50 (16,3)	18,50	6,34	17,30	6,64	13,40	5,38	9,56	3,75	58,76	21,02
	1 hour	76 (24,8)	19,32	4,91	17,84	5,31	13,29	4,53	10,07	3,42	60,51	16,66
	2 horas	64 (20,9)	18,97	5,87	16,98	6,12	12,73	4,72	9,67	3,69	58,36	18,98
	3 horas	27 (8,8)	18,41	3,65	17,44	4,01	12,63	3,41	9,52	2,55	58,00	12,16
	≥4 horas	16 (5,2)	21,69	7,51	19,94	6,56	14,88	5,33	10,19	3,10	66,69	20,92
	Test y significancia		F = 0,973 p = 0,451		F = 0,720 p = 0,655		F = 0,751 p = 0,629		F = 0,431 p = 0,882		F = 0,754 p = 0,626	
Absentismo escolar para jugar a juegos digitales	Si	5 (1,6)	13,40	2,61	12,20	4,15	10,40	4,34	6,40	1,14	42,40	10,36
	No	301 (98,4)	19,08	5,96	17,61	6,12	13,20	5,01	9,81	3,57	59,70	19,39
	Test y significancia		t = −2,124 p = 0,034		t = −1,969 p = 0,050		t = −1,241 p = 0,216		t = −6,203 p = 0,001		t = −1,988 p = 0,048	
Si la madre juega a juegos digitales	Si	78 (25,5)	20,03	5,48	18,26	5,67	13,50	4,40	10,29	3,19	62,08	17,54
	No	228 (74,5)	18,64	6,10	17,28	6,27	13,03	5,20	9,57	3,68	58,51	19,94
	Test y significancia		t = 1,782 p = 0,076		t = 1,220 p = 0,223		t = 0,715 p = 0,475		t = 1,551 p = 0,122		t = 1,403 p = 0,162	
Si el padre juega a juegos digitales	Si	87 (28,4)	18,60	5,76	16,74	5,24	12,39	4,03	9,43	3,29	57,15	16,75
	No	219 (71,6)	19,15	6,05	17,84	6,43	13,45	5,32	9,89	3,67	60,32	20,32
	Test y significancia		t = −0,725 p = 0,469		t = −1,425 p = 0,155		t = −1,888 p = 0,060		t = −1,018 p = 0,309		t = −1,293 p = 0,197	
Si la madre o el padre conocen el contenido de los juegos digitales a los que juega el niño.	Si	144 (47,1)	18,96	6,09	17,57	6,10	13,21	5,15	9,73	3,61	59,47	19,60
	No	162 (52,9)	19,02	5,87	17,49	6,18	13,10	4,89	9,78	3,54	59,38	19,27
	Test y significancia		t = −0,088 p = 0,930		t = 0,116 p = 0,907		t = 0,191 p = 0,849		t = −0,119 p = 0,906		t = 0,037 p = 0,970	
Si la madre o el padre juegan a juegos digitales con el niño	Si	44 (14,4)	19,95	5,49	18,27	5,96	13,66	5,39	10,43	3,66	62,32	19,44
	No	262 (85,6)	18,83	6,04	17,40	6,16	13,06	4,94	9,64	3,55	58,94	19,38
	Test y significancia		t = 1,159 p = 0,247		t = 0,873 p = 0,383		t = 0,728 p = 0,467		t = 1,361 p = 0,174		t = 1,071 p = 0,285	
Si la madre o el padre supervisan el juego digital	Si	195 (63,7)	18,62	5,78	17,23	6,06	12,93	5,03	9,70	3,71	58,48	19,33
	No	111 (36,3)	19,65	6,26	18,05	6,25	13,53	4,95	9,85	3,33	61,07	19,48
	Test y significancia		t = −1,459 p = 0,146		t = −1,118 p = 0,264		t = −1,006 p = 0,315		t = −0,339 p = 0,735		t = −1,124 p = 0,262	
Considerarse un adicto a los juegos digitales	Si	6 (2,0)	21,33	9,79	19,00	8,07	14,83	6,82	9,00	2,97	64,17	25,90
	Partly	43 (14,0)	18,72	5,37	17,98	5,68	13,79	4,51	9,98	3,58	60,47	17,97
	No	257 (84,0)	18,98	5,97	17,42	6,17	13,00	5,04	9,74	3,59	59,14	19,53
	Test y significancia		F = 0,505 p = 0,604		F = 0,330 p = 0,719		F = 0,801 p = 0,450		F = 0,220 p = 0,803		F = 0,268 p = 0,765	

DE: Desvío estándar.

La diferencia entre las puntuaciones de los niños en las subdimensiones del desarrollo de la tolerancia durante el tiempo de juego y el valor otorgado al juego, la excesiva atención a los juegos digitales y los conflictos, y el aplazamiento de los deberes/tareas individuales y sociales y el DGASC total fue estadísticamente significativa en función del sexo de los padres. Las puntuaciones medias de las subdimensiones y totales de los hijos de padres de sexo femenino fueron significativamente más altas (p < 0,05). La diferencia entre las puntuaciones medias de los alumnos en las subdimensiones excesiva concentración en jugar a juegos digitales y conflicto, postergación de deberes/tareas individuales y sociales, y reflejo psicológico-fisiológico de privación e inmersión en el juego y la DGASC total fue estadísticamente significativa según la frecuencia de uso de dispositivos tecnológicos por parte de los padres y los dispositivos tecnológicos que utilizaban. En el análisis posterior realizado para determinar qué grupo causaba la diferencia en cuanto a la frecuencia de uso de dispositivos tecnológicos, se determinó que las puntuaciones medias de la escala de los niños de aquellos que utilizaban dispositivos tecnológicos varias veces al mes eran superiores a las de los demás grupos (p < 0,05) (Tabla 4).

**Tabla 4:** Comparación de las puntuaciones DGASC y subdimensiones según las características demográficas de los padres – Ankara, Turquía, 2024.

Características sociodemográficas		n (%)	Desarrollo de la tolerancia durante el recreo y valor del juego		Concentración excesiva en los juegos digitales y los conflictos		Aplazamiento de deberes/tareas individuales y sociales		Reflejo psicológico-fisiológico de la privación y la inmersión en el juego		DGASC	
			Mean	SD	Mean	SD	Mean	SD	Mean	SD	Mean	SD
Género	Masculino	99 (32,4)	17,71	5,98	16,36	5,94	12,31	4,47	9,27	3,23	55,66	18,43
	Femenino	207 (67,6)	19,60	5,88	18,08	6,15	13,55	5,20	9,99	3,71	61,22	19,63
	Test y significancia		t = −2,626 p = 0,009		t = −2,311 p = 0,021		t = -2,035 p = 0,043		t = -1,639 p = 0,102		t = -2,366 p = 0,019	
Edades	25–29	2 (0,7)	14,50	3,54	15,00	4,24	9,00	4,24	7,00	1,41	45,50	13,44
	30–34	65 (21,2)	20,74	5,79	19,52	5,82	14,25	5,01	10,86	3,42	65,37	18,89
	35–39	119 (38,9)	18,73	5,84	17,38	6,25	12,99	4,91	9,75	3,73	58,85	19,59
	40–44	73 (23,8)	18,51	6,05	16,70	6,11	13,00	5,26	9,25	3,47	57,45	19,37
	45–49	32 (10,5)	17,97	5,14	16,44	4,99	12,50	3,93	9,06	3,19	55,97	15,64
	49+	15 (4,9)	18,60	8,09	16,73	7,67	12,33	6,31	9,33	3,62	57,00	24,43
	Test y significancia		F = 1,703 p = 0,134		F = 2,014 p = 0,077		F = 1,127 p = 0,346		F = 2,104 p = 0,065		F = 1,876 p = 0,098	
Tipo familiar	Extendida	31 (10,1)	18,65	5,90	16,94	5,43	12,29	3,22	9,58	3,49	57,45	16,81
	Nuclear	265 (86,6)	19,03	5,95	17,61	6,18	13,25	5,13	9,78	3,57	59,67	19,57
	Rota	10 (3,3)	19,10	7,17	17,10	7,25	13,10	6,26	9,60	4,22	58,90	23,53
	Test y significancia		F = 0,058 p = 0,944		F = 0,193 p = 0,825		F = 0,513 p = 0,599		F = 0,053 p = 0,948		F = 0,185 p = 0,832	
Educación	Analfabeto	5 (1,6)	19,00	6,48	18,00	6,96	13,60	6,54	9,80	3,90	60,40	23,42
	Escuela primaria	46 (15,0)	19,80	5,08	17,54	5,30	13,46	3,87	9,59	3,36	60,39	15,83
	Secundaria	57 (18,6)	19,30	6,18	17,91	6,67	13,05	5,35	9,89	3,48	60,16	20,42
	Bachillerato	119 (38,9)	18,71	6,29	17,34	6,29	13,13	4,95	9,81	3,80	58,97	20,14
	Universitario	75 (24,5)	18,57	5,87	17,59	6,16	13,05	5,55	9,68	3,54	58,89	20,10
	Postgrado	4 (1,3)	21,50	5,57	15,75	0,50	13,00	2,16	9,50	1,00	59,75	2,99
	Test y significancia		F = 0,466 p = 0,801		F = 0,141 p = 0,983		F = 0,053 p = 0,998		F = 0,053 p = 0,998		F = 0,065 p = 0,997	
Número de hijos	1	62 (20,3)	18,42	6,07	17,71	6,17	12,60	5,34	9,71	3,80	58,44	20,21
	2	164 (53,6)	19,18	5,67	17,68	6,04	13,51	4,86	9,87	3,51	60,23	18,73
	3	57 (18,6)	18,39	7,03	16,44	6,86	12,63	5,43	9,40	3,87	56,86	22,01
	4	21 (6,9)	21,29	4,28	19,14	4,22	13,81	3,71	10,24	2,59	64,48	13,38
	≥5	2 (0,7)	14,00	5,66	13,50	6,36	9,00	4,24	7,00	1,41	43,50	17,68
	Test y significancia		F = 1,466 p = 0,212		F = 1,070 p = 0,372		F = 0,985 p = 0,416		F = 0,571 p = 0,684		F = 1,055 p = 0,379	
Nivel económico	Ingresos<gastos	28 (9,2)	18,29	5,29	16,54	5,97	12,82	4,60	9,64	4,05	57,29	18,72
	Ingresos=gastos	248 (81,0)	19,16	5,97	17,79	6,01	13,21	4,97	9,87	3,48	60,02	19,12
	Ingresos>gastos	30 (9,8)	18,27	6,63	16,30	7,18	12,97	5,76	8,93	3,86	56,47	22,32
	Test y significancia		F = 0,511 p = 0,600		F = 1,191 p = 0,305		F = 0,098 p = 0,907		F = 0,930 p = 0,396		F = 0,635 p = 0,531	
Trabajo	Ama de Casa	145 (47.4)	19.62	5.91	18.10	6.32	13.70	5.40	10.03	3.73	61.45	20.14
	Empleado público	42 (13.7)	18.00	6.16	17.79	6.28	13.14	5.05	9.98	3.63	58.90	20.26
	Trabajador	31 (10.1)	8.97	5.49	6.52	5.30	2.42	3.61	9.87	3.28	57.77	5.62
	Autónomo	20 (6,5)	18,20	7,52	15,70	7,12	12,05	5,03	9,05	3,63	55,00	22,43
	Comerciante	17 (5,6)	17,82	5,73	17,35	6,08	12,76	5,09	9,88	4,37	57,82	20,09
	Sector privado	35 (11,4)	18,54	4,88	17,09	5,52	12,66	4,75	8,94	3,13	57,23	17,28
	Otro	16 (5,2)	19,13	7,38	17,06	5,76	12,44	3,88	9,00	2,16	57,63	17,63
	Test y significancia		F = 0,659 p = 0,683		F = 0,702 p = 0,648		F = 0,690 p = 0,658		F = 0,724 p = 0,630		F = 0,591 p = 0,737	
Frecuencia de uso de dispositivos tecnológicos	Casi nunca	21 (6,9)	16,90	6,60	14,29	5,94	11,57	4,70	8,38	3,58	51,14	19,13
	Algunas veces al mes	5 (1,6)	22,60	5,68	24,20	5,07	19,20	4,97	13,20	2,68	79,20	17,57
	Varias veces a la semana	15 (4,9)	17,00	5,04	13,93	5,50	11,60	4,29	8,33	3,29	50,87	16,73
	Casi diariamente	233 (76,1)	19,24	5,99	17,87	6,10	13,27	5,07	9,91	3,59	60,28	19,51
	Casi siempre	32 (10,5)	18,94	5,49	17,78	5,42	13,13	4,37	9,66	3,22	59,50	17,26
	Test y significancia		F = 1,629 p = 0,167		F = 4,650 p = 0,001		F = 2,808 p = 0,026		F = 2,712 p = 0,030		F = 3,191 p = 0,014	
Competencia en el uso de herramientas tecnológicas	Muy buena	12 (3,9)	19,00	6,02	17,00	6,44	12,00	5,38	9,08	3,42	57,08	20,14
	Buena	140 (45,8)	18,53	5,63	17,28	5,91	12,96	4,84	9,61	3,45	58,39	18,61
	Moderada	130 (42,5)	19,36	6,39	17,73	6,44	13,36	5,19	9,92	3,80	60,37	20,42
	Baja	21 (6,9)	20,00	5,54	18,48	5,97	13,76	5,18	9,95	3,38	62,19	19,34
	Incompetente	3 (1,0)	17,33	6,43	15,67	4,16	13,00	2,65	10,67	1,15	56,67	13,05
	Test y significancia		F = 0,540 p = 0,706		F = 0,308 p = 0,873		F = 0,341 p = 0,850		F = 0,288 p = 0,885		F = 0,340 p = 0,851	
Jugar a juegos digitales	Si	101 (33,0)	19,52	5,61	18,35	5,84	13,75	5,13	10,13	3,51	61,75	18,86
	No	205 (67,0)	18,73	6,13	17,12	6,24	12,85	4,92	9,57	3,59	58,27	19,60
	Test y significancia		t = 1,100 p = 0,272		t = 1,648 p = 0,100		t = 1,481 p = 0,140		t = 1,481 p = 0,140		t = 1,479 p = 0,140	
Tiempo medio diario dedicado a los juegos digitales	Nada	203 (66,3)	18,77	6,15	17,17	6,27	12,87	4,95	9,59	3,61	58,39	19,69
	0–15 minutos	10 (3,3)	17,70	6,04	14,90	4,98	10,60	3,69	8,50	2,72	51,70	15,63
	15–30 minutos	20 (6,5)	19,90	6,33	18,40	7,76	14,50	6,18	10,15	4,15	62,95	22,97
	30 minutos–1 hora	33 (10,8)	19,36	5,86	18,82	5,78	14,06	5,25	10,45	3,46	62,70	19,55
	1 hora	18 (5,9)	19,83	3,47	18,67	3,56	13,50	3,03	10,00	3,18	62,00	11,45
	2 horas	16 (5,2)	20,69	5,61	19,38	5,29	14,19	5,00	11,19	3,37	65,44	18,26
	3 horas	4 (1,3)	17,75	8,06	16,75	6,65	15,00	7,75	8,50	3,42	58,00	25,10
	≥4 horas	2 (0,7)	14,00	2,83	13,00	5,66	11,00	7,07	6,50	0,71	44,50	16,26
	Test y significancia		F = 0,647 p = 0,717		F = 1,091 p = 0,369		F = 1,072 p = 0,381		F = 1,150 p = 0,332		F = 0,974 p = 0,450	
Tiempo medio que el niño dedica al día a juegos digitales	Nada	38 (12,4)	16,53	6,72	14,74	6,42	10,84	4,84	8,42	3,98	50,53	21,04
	0–15 minutos	13 (4,2)	15,92	4,61	16,31	6,34	13,31	5,53	9,00	3,70	54,54	18,34
	15–30 minutos	18 (5,9)	18,78	7,90	16,67	7,59	12,89	6,32	9,06	4,08	57,39	24,72
	30 minutos–1 hora	54 (17,7)	19,76	5,67	18,80	5,79	13,65	5,19	10,50	3,54	62,70	18,92
	1 hora	79 (25,8)	19,22	5,28	17,77	5,34	13,16	3,99	9,97	2,87	60,13	16,16
	2 horas	66 (21,6)	19,85	5,66	18,55	6,06	13,82	5,24	10,12	3,78	62,33	19,58
	3 horas	24 (7,8)	20,08	5,94	17,04	6,24	13,71	5,16	9,88	3,83	60,71	19,91
	≥4 horas	14 (4,6)	18,64	6,95	17,07	7,25	13,50	5,53	8,93	3,34	58,14	21,32
	Test y significancia		F = 1,920 p = 0,066		F = 1,932 p = 0,064		F = 1,478 p = 0,175		F = 1,548 p = 0,151		F = 1,792 p = 0,088	

DE: Desvío estándar.

No hubo relación significativa entre las puntuaciones medias en las subdimensiones de la DGASC total y desarrollo de la tolerancia durante el tiempo de juego y el valor otorgado al juego, excesiva concentración en jugar a juegos digitales y conflicto, postergación de deberes/tareas individuales y sociales, y reflejo psicológico-fisiológico de privación e inmersión en el juego y la DPAS total y subdimensiones (p > 0,05) (Tabla 5). Al no existir relación entre las puntuaciones totales de ambas escalas, no se realizó análisis de regresión.

**Tabla 5:** Examen de la correlación entre DPAS y DGASC – Ankara, Turquía, 2024.

		Aprobación del uso eficaz de los medios digitales	Protección frente a los riesgos de los medios digitales	DPAS
Desarrollo de la tolerancia durante el recreo y valor del juego	r	0,011	-0,097	-0,051
	p	0,844	0,089	0,372
Concentración excesiva en los juegos digitales y los conflictos	r	0,023	-0,074	-0,031
	p	0,693	0,197	0,591
Aplazamiento de deberes/tareas individuales y sociales	r	0,003	-0,074	-0,042
	p	0,958	0,194	0,461
Reflejo psicológico-fisiológico de la privación y la inmersión en el juego	r	0,017	-0,077	-0,055
	p	0,773	0,182	0,342
DGASC	r	0,008	-0,087	-0,046
	p	0,884	0,131	0,418

## DISCUSIÓN

La investigación del efecto de las actitudes parentales digitales sobre la adicción a los juegos digitales es fundamental para tener algunas ideas sobre los efectos del creciente uso de juegos digitales en los estudiantes de hoy en día. Los estudiantes que juegan a juegos digitales sin control corren el riesgo de sufrir adicción a los juegos digitales^(4)^. Según los resultados de nuestra investigación, el 83% de los estudiantes y el 33% de sus padres que participaron en la investigación declararon que jugaban a juegos digitales. Más de la mitad de los estudiantes jugaban a juegos digitales al menos una hora al día, casi la mitad de ellos declararon que sus padres no sabían nada sobre el contenido de los juegos digitales a los que jugaban, y el 36,3% declaró que sus padres no controlaban su forma de jugar. Además, el 59,8% de los padres declararon que sus hijos jugaban a juegos digitales una media de al menos una hora al día. Los juegos digitales atraen la atención de personas de todas las edades, pero el aumento del tiempo que los estudiantes dedican a los juegos digitales demuestra que corren el riesgo de caer en la adicción a los juegos digitales. Empezar a jugar a juegos digitales a una edad temprana, jugar sin supervisión y que los padres jueguen a juegos digitales aumentan el riesgo de adicción. Conocer estos riesgos es muy importante para tomar medidas preventivas^(2,22)^. Según los resultados de nuestra investigación, puede decirse que los estudiantes pasan mucho tiempo delante de la pantalla jugando a juegos digitales. De forma similar a los resultados de nuestro estudio, en la literatura nacional e internacional se informa de que el número de niños que juegan a juegos digitales es bastante elevado. Según la investigación realizada por el Instituto Turco de Estadística (TURKSTAT), el 82,7% de los niños de entre 6 y 15 años utilizaban Internet, y el 36% de los que lo hacían jugaban a juegos digitales. Según esta investigación de TURKSTAT, el 94,7% de los niños de entre 6 y 15 años que declararon jugar a juegos digitales lo hacían con regularidad, casi todos los días o al menos una vez a la semana^(23)^. Según el informe «Newzoo 2020 Global Games Market Report», el número de jugadores de juegos digitales en todo el mundo alcanzó los 3.200 millones y se espera que llegue a los 3.500 millones en 2025^(24)^. Jugar a juegos digitales se ha convertido hoy en algo casi inevitable. En este punto, considerar los riesgos y las posibilidades, y regular su uso de acuerdo con la edad y las necesidades del niño es una de las responsabilidades importantes de los padres^(25)^. Las enfermeras pueden monitorear los efectos de los juegos digitales en los niños e informar a las familias y a los estudiantes para un uso equilibrado. Pueden concienciar sobre el tiempo de juego, la salud física y los efectos psicológicos, y fomentar hábitos de juego saludables. Es importante garantizar que los niños utilicen los juegos digitales de forma segura y educativa colaborando con las familias. Las enfermeras pueden ayudar a crear políticas sobre el uso de los juegos digitales en las escuelas para que los niños puedan divertirse y continuar su desarrollo saludable. Este enfoque pretende reducir los efectos negativos de los juegos digitales al tiempo que apoya sus aspectos positivos. Hay varias situaciones importantes entre los diagnósticos de enfermería que las enfermeras de salud escolar pueden encontrar en relación con el uso de los juegos digitales. La falta de actividad física, jugar durante largos periodos de tiempo, la inactividad y los trastornos posturales pueden conducir al riesgo de obesidad. Tensión ocular, pasar largas horas delante de la pantalla puede provocar molestias como dolor, ardor y visión borrosa en los ojos. Trastornos del sueño, jugar hasta altas horas de la noche puede alterar los patrones de sueño de los niños y causar problemas como insomnio y despertarse tarde. Aislamiento social, el uso excesivo de juegos digitales puede impedir las interacciones sociales de los niños, lo que puede provocar deficiencias en las habilidades sociales. Estrés emocional y psicológico, los juegos violentos o los fracasos pueden provocar problemas emocionales como ansiedad y depresión. Por último, la sobrecarga mental, es una situación en la que el uso excesivo de juegos puede causar distracción y una disminución del éxito académico. Las enfermeras pueden tener en cuenta estos diagnósticos y realizar intervenciones^(11,26,27)^. En nuestra investigación, la puntuación media del DGASC fue de 59,42 puntos. Teniendo en cuenta esta puntuación media, los estudiantes se encontraban en el grupo de riesgo de adicción a los juegos digitales. Según las evaluaciones de la adicción a los juegos digitales realizadas en nuestra investigación, el 14,4% de los alumnos eran «adictos», el 2,6% eran «muy adictos» y el 54,9% se encontraban en el grupo de «riesgo».

De acuerdo con nuestros hallazgos, podemos decir que los juegos digitales son una actividad popular en la vida de los estudiantes de secundaria, pero crean un riesgo de adicción para los estudiantes. En un estudio similar en la literatura, se afirmó que los juegos digitales eran una actividad popular para los estudiantes^(28)^. En este estudio, realizado con 1.046 alumnos de enseñanza media, se observó que la puntuación media DGASC (47,74) se situaba en un nivel normal para la adicción a los juegos digitales. También afirmaron que el 7,4% de sus participantes eran «adictos», el 1,5% eran «muy adictos» y el 33,9% estaban en el grupo de «riesgo». Según el Instituto Turco de Estadística, la tasa de uso de las tecnologías de la información en niños era del 50,8% en 2013 y alcanzaba el 82,7% en 2021, lo que revela la probabilidad de un aumento crítico de la tasa de individuos adictos que puede encontrarse en los próximos años^(23)^. Por lo tanto, las intervenciones necesarias en relación con los efectos de la creciente adicción a los juegos son de vital importancia no sólo para aquellos que son adictos a los juegos, sino para todos los estudiantes. Las intervenciones también deberían centrarse en prevenir la adicción a los juegos en los niños que aún no la padecen. Las prácticas preventivas deben incluir el intercambio de información sobre la adicción a los juegos, los peligros potenciales de la exposición excesiva a la tecnología y los juegos apropiados para el contenido, así como limitar el tiempo de pantalla a menos de una hora al día. Deben apoyarse los programas educativos para prevenir la adicción a los juegos digitales, y deben desarrollarse políticas para limitar el uso de dispositivos electrónicos personales durante la educación formal. Para los niños con adicción a los juegos, también deberían utilizarse métodos como la terapia cognitivo-conductual y la terapia familiar en el tratamiento de la adicción a los juegos digitales^(10)^. En un centro escolar en el que los alumnos corren el riesgo de sufrir adicción a los juegos digitales, las enfermeras pueden tomar varias precauciones. En primer lugar, pueden concienciar a los alumnos sobre los perjuicios de la adicción a los juegos. Pueden sugerir límites y un uso equilibrado del tiempo frente a la pantalla. También pueden ayudar a las familias a desarrollar hábitos saludables en casa, orientándolas sobre la duración y el contenido de los juegos. Las enfermeras pueden organizar actividades de grupo y fomentar la actividad física para que los alumnos desarrollen habilidades de interacción social. Pueden realizar entrevistas individuales con los alumnos que muestren signos de adicción y proporcionarles derivaciones donde puedan recibir apoyo profesional cuando sea necesario. Este enfoque fomenta hábitos de juego saludables^(26,27)^.

En nuestro estudio, la diferencia entre la puntuación media total del DGASC según la variable de faltar a clase para jugar a juegos digitales resultó ser estadísticamente significativa. Las puntuaciones medias de los alumnos que no faltaron a clase fueron superiores. Cuando se examinó la bibliografía, se vio que la adicción a los juegos digitales causaba fracaso escolar^(29,30)^. Contrariamente a lo que se afirma en la bibliografía, la razón por la que las puntuaciones medias de los alumnos que no faltaron a clase en la escala de adicción a los juegos digitales fueron significativamente más altas en nuestros resultados puede deberse al interés de los alumnos por los juegos digitales. El interés de los alumnos que no faltaron a clase por los juegos digitales puede haberse desarrollado más como resultado de sus interacciones con sus amigos. Además, los alumnos que iban al colegio con regularidad pueden haber aprendido a gestionar mejor su tiempo, pero pueden haber pasado la mayor parte de su tiempo libre después del colegio jugando a juegos digitales. Otra razón puede haber sido que las dificultades que conlleva la vida escolar hicieron que los estudiantes prefirieran los juegos digitales como medio de evasión. Las enfermeras pueden tomar varias iniciativas contra la adicción de los estudiantes a los juegos digitales. En primer lugar, pueden concienciar informando a los estudiantes y a las familias sobre los efectos de los juegos en el éxito académico. También pueden orientar sobre el desarrollo de habilidades de gestión del tiempo y ayudar a los estudiantes a equilibrar sus actividades extraescolares. Las enfermeras pueden organizar actividades alternativas divertidas y relajantes para ayudar a los estudiantes a hacer frente al estrés. Pueden realizar entrevistas individuales con los estudiantes que utilizan los juegos digitales como herramienta de evasión y orientarles hacia un apoyo profesional. Estas iniciativas pueden ayudar a los estudiantes a desarrollar hábitos saludables. La inactividad, los trastornos nutricionales, la obesidad, los trastornos mentales y muchos otros problemas surgen debido a la adicción a los juegos digitales. Los adolescentes adictos a los juegos digitales no hacen ejercicio, y el aumento del tiempo que pasan delante del juego provoca muchos problemas, como trastornos musculoesqueléticos, obesidad, enfermedades oculares y trastornos nutricionales. Jugar durante mucho tiempo también puede desencadenar alteraciones en la vida social, alejamiento de los amigos y un estilo de vida asocial. A medida que aumenta el tiempo dedicado a los juegos, aumenta el número de deberes olvidados y disminuye el nivel de éxito académico^(12,13)^.

Se observó que las puntuaciones de los alumnos de octavo curso de la subdimensión «postergación de deberes/tareas individuales y sociales», que es uno de los subfactores de la escala de adicción a los juegos digitales, eran significativamente más altas. Del mismo modo, en la bibliografía, los estudios sobre el examen de la relación entre la adicción a los juegos digitales de los alumnos de enseñanza media y sus datos demográficos indicaron que los alumnos de octavo curso presentaban niveles de adicción a los juegos digitales más elevados que los de otros cursos^(31,32)^. Otro estudio realizado por Gül y Özgür mostró una diferencia significativa entre los niveles de adicción a los juegos digitales de los alumnos y su curso^(28)^. En la bibliografía se destacaba que el uso de pantallas aumentaba a medida que el niño crecía y que la gran mayoría de los niños pasaban tiempo frente a una pantalla muy por encima de los límites recomendados por la Asociación Americana de Pediatría^(25)^. Las razones por las que los niveles de adicción de los alumnos aumentan a medida que aumenta el grado escolar fueron que es posible que se hayan independizado más de sus familias, se hayan vuelto más autónomos y socializados con el aumento de su edad y el efecto de la pubertad, y que hayan recurrido más a los entornos virtuales y, por tanto, a los juegos digitales, que son una actividad popular entre los niños, debido a su deseo de ser aceptados en un grupo. Además, la razón por la que los alumnos de8º curso presentaban mayores niveles de adicción a los juegos digitales que los de 5º, 6º y 7º puede deberse a que los alumnos de este grupo de edad están más familiarizados con la tecnología y juegan más a juegos digitales con sus amigos debido al desarrollo de sus círculos sociales.

La diferencia entre las puntuaciones medias totales del DGASC según el sexo de los padres fue estadísticamente significativa. Las puntuaciones medias de los hijos de padres de sexo femenino fueron significativamente más altas. El examen de los datos demográficos de los padres en nuestro estudio indicó que la mayoría de los responsables eran madres (67,6%). Nuestros hallazgos mostraron que los hijos que eran cuidados por madres tenían puntuaciones más altas de adicción a los juegos digitales que las puntuaciones de los hijos cuyos responsables eran padres. Doğan y Döğer hallaron una relación positiva entre el tiempo que pasaban diariamente en Internet las madres y sus hijos^(19)^. Teniendo en cuenta que las madres suelen pasar más tiempo comunicándose con sus hijos como progenitoras que los padres, el tiempo que las madres dediquen a los dispositivos tecnológicos será tiempo libre para el niño. Es un resultado esperado que el niño dedique tiempo a los dispositivos tecnológicos como consecuencia de tomar a la madre como modelo durante este tiempo. El tiempo que las madres que priorizan el cuidado de sus hijos pasan en Internet afecta a los niños^(19,33)^. Las enfermeras pueden desarrollar estrategias de intervención teniendo en cuenta el uso que hacen los padres de los medios digitales y sus efectos en los niños. Las enfermeras pueden organizar programas de concienciación dirigidos a evitar que las madres aumenten su uso de la tecnología y proporcionar a las madres formación sobre la adicción digital. También pueden orientar a las familias para que los niños utilicen la tecnología de forma saludable reforzando la comunicación con los padres. Las enfermeras pueden animar a las madres a pasar más tiempo de calidad con sus hijos y a equilibrar la tecnología con la interacción social y las actividades físicas. De este modo, pueden ayudar a los niños a adquirir hábitos saludables reduciendo su riesgo de adicción a los juegos digitales^(11)^.

La diferencia entre las puntuaciones medias totales del DGASC fue estadísticamente significativa según la frecuencia con la que los padres utilizaban dispositivos tecnológicos. En el análisis avanzado realizado para determinar qué grupo causaba la diferencia según la frecuencia de uso de los dispositivos tecnológicos, se observó que las puntuaciones medias de la escala de los niños cuyos padres utilizaban estos dispositivos varias veces al mes eran superiores a las puntuaciones de los demás grupos. En un estudio sobre la relación entre padres e hijos en cuanto a la adicción a internet, Doğan y Döğer concluyeron que existía una relación significativa entre el tiempo que los padres y sus hijos dedicaban diariamente a los dispositivos tecnológicos^(19)^. Los padres son modelos para sus hijos en el uso de la tecnología. Cuando los padres se comunican menos cara a cara con sus hijos, estos pueden tender a recurrir más a los juegos digitales. Como resultado de esta situación, puede aumentar la adicción a los juegos digitales. Dado que los padres son los modelos más cercanos para sus hijos, sus acciones tienen un gran efecto en ellos. Los niños cuyos padres juegan a juegos digitales corren un mayor riesgo de convertirse en adictos a estos juegos^(28)^. Es importante que las enfermeras desarrollen diversas estrategias para los hábitos de uso de la tecnología de los padres. Las enfermeras pueden educar a los padres sobre los efectos del uso de los medios digitales en los niños y orientarles sobre cómo equilibrar el interés de los niños por la tecnología. También pueden llevar a cabo actividades de concienciación para que los padres limiten el uso de la tecnología y se comuniquen más cara a cara. Pueden proporcionar orientación haciendo hincapié en que los padres deben equilibrar el tiempo que utilizan los medios digitales, sin olvidar que son modelos de conducta para sus hijos. Al concienciar a las familias sobre este tema, las enfermeras pueden ayudar a los niños a adquirir hábitos saludables para prevenir la adicción a los juegos digitales^(11)^.

En el estudio, la puntuación media en la escala total de actitudes de los padres hacia los medios digitales fue de 46,97. El rápido aumento del uso de las tecnologías digitales por parte de los niños llama la atención sobre la importancia de las actitudes de los padres hacia estas tecnologías. Es necesario concienciar a los padres para que los niños puedan beneficiarse de las oportunidades de aprendizaje que ofrecen las tecnologías digitales y estar protegidos de sus riesgos^(4)^. Es importante evaluar las actitudes de los padres para que los niños puedan beneficiarse de las oportunidades de la tecnología a través de sus padres y estar protegidos de sus riesgos. Esto se debe a que las actitudes son marcos cognitivos y afectivos y se consideran determinantes permanentes y consistentes en la predicción del comportamiento^(4)^. Por este motivo, los padres deben ser capaces de utilizar roles parentales digitales apropiados para crear y gestionar entornos seguros y adecuados a las necesidades de aprendizaje de sus hijos, y deben ser capaces de mostrar actitudes que sean eficaces a la hora de elegir estos roles. Las actitudes parentales digitales de alto nivel de los padres de hoy en día han cobrado gran importancia a la hora de concienciarlos sobre los beneficios y los riesgos de la tecnología, tomar las precauciones necesarias al respecto y ser capaces de guiar a sus hijos. Las actitudes de los padres tienen un gran impacto en el desarrollo de los niños, tanto social como cognitivo. En este punto, las actitudes de los padres también deben ser de apoyo^(34)^. Las enfermeras pueden organizar programas educativos que expliquen a los padres los efectos, beneficios y riesgos potenciales de las tecnologías digitales en los niños. También es importante organizar formaciones periódicas para los padres con el fin de mejorar las conductas de salud de los adolescentes y adquirir comportamientos de juego saludables^(12)^. Cabe destacar que los padres deben desarrollar una actitud consciente y de apoyo para garantizar que sus hijos estén seguros en el mundo digital. También pueden concienciar sobre la crianza digital y orientar a los padres para que sus hijos establezcan relaciones sanas con los juegos y las tecnologías digitales. Conocer los factores que afectan a la adicción de los adolescentes a los juegos es muy importante para que las enfermeras pediátricas puedan identificar a los adolescentes que corren riesgo de adicción a los juegos y tomar las precauciones necesarias^(12,26,27)^.

En el estudio, se examinó la relación entre la Escala de Actitudes Digitales de los Padres y la Escala de Adicción a los Juegos Digitales para Niños y no se encontró ninguna relación significativa. Contrariamente a nuestros hallazgos, algunos estudios en la literatura han demostrado que las actitudes parentales están relacionadas con los niveles de adicción a los juegos de los niños^(19,25,35-37)^. La razón por la que no encontramos una relación significativa entre las actitudes parentales digitales y la adicción a los juegos digitales de los estudiantes en este estudio puede haber sido que los padres de nuestro grupo de estudio no habían sido informados sobre los daños potenciales de los juegos digitales. Según los resultados de nuestro estudio, casi todos los estudiantes y el 33% de los padres declararon que jugaban a juegos digitales. Además, más de la mitad de los alumnos jugaban a juegos digitales al menos una hora al día, casi la mitad de los alumnos declararon que sus padres no sabían nada sobre el contenido de los juegos digitales a los que jugaban, y el 36,3% declaró que sus padres no les controlaban mientras jugaban a juegos digitales. Todos estos hallazgos pueden sugerir que los padres tenían poca conciencia y un control inadecuado sobre el juego digital de sus hijos, no guiaban a sus hijos en los juegos digitales e ignoraban los posibles riesgos en el mundo digital. Al apoyar las actitudes de los padres hacia el uso de los medios digitales, las enfermeras pueden ayudar a los niños a desarrollar hábitos saludables que los mantendrán seguros en el mundo digital. Las enfermeras pueden evaluar las conductas de juego de los estudiantes tanto en la práctica clínica como en la investigación y aprovechar los programas de intervención digital en línea dirigidos a mejorar las estrategias y habilidades de afrontamiento^(27)^.

## CONCLUSIÓN

En este estudio, en el que investigamos los efectos de las actitudes parentales digitales sobre la adicción a los juegos digitales en estudiantes de secundaria, se descubrió que aproximadamente tres cuartas partes de los estudiantes jugaban a juegos digitales y que más de la mitad jugaba a estos juegos durante al menos una hora al día. Cuando se evaluó a los estudiantes según sus niveles de adicción a los juegos digitales, se determinó que aproximadamente tres cuartas partes eran adictos a los juegos o se encontraban en el grupo de riesgo. No se encontró ninguna relación significativa entre las actitudes de los padres hacia los juegos digitales y los niveles de adicción a los juegos digitales de los estudiantes.

Las enfermeras pueden proporcionar apoyo a padres y estudiantes para reducir el riesgo de adicción a los juegos digitales en los niños. Es muy importante organizar programas de formación sobre la adicción a los juegos digitales para los padres y hacer hincapié en que deben ser modelos positivos para sus hijos en estos programas. Los enfermeros pueden orientar a los padres a la hora de poner límites a los juegos digitales de sus hijos, concienciarlos sobre los comportamientos seguros en línea y monitorear los hábitos de juego de sus hijos. Además, pueden concienciar a los estudiantes sobre este problema organizando programas sobre la adicción a los juegos digitales. Las pruebas de detección de la adicción a los juegos digitales también deberían incluirse en los programas periódicos de exámenes de salud realizados por la enfermera. Las enfermeras pueden apoyar a los estudiantes adictos a los juegos digitales y en situación de riesgo manteniendo entrevistas individuales y pueden asegurarse de que reciban ayuda profesional cuando sea necesario. Pueden enseñar a los estudiantes estrategias preventivas como la gestión del tiempo y el afrontamiento del estrés. Es muy importante añadir lecciones de protección y prevención al currículo educativo para prevenir la adicción a los juegos digitales. La adicción a los juegos digitales es común entre los estudiantes. Según nuestros resultados, aproximadamente tres cuartas partes de los estudiantes son adictos a los juegos o se encuentran en el grupo de riesgo. Las enfermeras deberían desarrollar estrategias para prevenir la adicción a los juegos digitales. Las actitudes digitales de los padres deben tenerse en cuenta al examinar la adicción a los juegos digitales en los estudiantes. Las enfermeras deberían organizar programas de formación para estudiantes y padres para prevenir la adicción a los juegos digitales.

## IMPLICACIONES PARA LA PRÁCTICA DE ENFERMERÍA

Las enfermeras deben detectar precozmente la adicción a los juegos digitales y estar preparadas para intervenir, y deben ser capaces de evaluar sus riesgos y síntomas. Deben utilizar escalas válidas y fiables para detectar la adicción a los juegos digitales en los estudiantes. Las enfermeras deben planificar programas de formación para profesores, alumnos y padres sobre la adicción a los juegos digitales. Se pueden crear programas escolares de intervención para prevenir y reducir la adicción a los juegos digitales. Los estudiantes adictos a los juegos digitales y en riesgo de adicción a los juegos deberían recibir ayuda profesional. Además, nuestros hallazgos guiarán a las enfermeras en la planificación de intervenciones. Nuestra investigación reveló que aproximadamente tres cuartas partes de los estudiantes eran adictos a los juegos o se encontraban en el grupo de riesgo. El personal de enfermería debería tener en cuenta esta situación y elaborar planes para reducir el nivel de adicción a los juegos colaborando con los alumnos, los padres y los profesores de orientación y asesoramiento psicológico de los centros escolares. En este contexto, deberían planificarse proyectos de concienciación que incluyan a profesores, alumnos y padres.

## PUNTOS FUERTES Y LIMITACIONES

Los datos de este estudio se recolectaron tanto de padres como de alumnos para revelar el efecto de las actitudes digitales de los padres sobre la adicción a los juegos digitales en alumnos de enseñanza media. Esta situación constituyó el punto fuerte del estudio, ya que aumentó la fiabilidad de los datos recolectados. Los datos se limitaron a las respuestas de 306 estudiantes de una escuela media estatal y 306 padres en el XX. Por lo tanto, los resultados obtenidos en el estudio sólo pueden generalizarse a este grupo.

## DISPONIBILIDAD DE DATOS

Los datos están disponibles a través del autor correspondiente previa solicitud razonable.
